# Cross-cultural beliefs and practices of caregivers regarding sunlight exposure for newborn jaundice: a scoping review

**DOI:** 10.3389/fpubh.2026.1888701

**Published:** 2026-08-19

**Authors:** Bolajoko O. Olusanya, Zainab O. Imam, Evans O. Asowata, Oluwatobi I. Olusanya, Abieyuwa A. Emokpae

**Affiliations:** 1 Centre for Healthy Start Initiative, Lagos, Nigeria; 2 Massey Street Children’s Hospital, Lagos, Nigeria

**Keywords:** heliotherapy, maternal beliefs, maternal knowledge, maternal practice, neonatal jaundice, newborn care, phototherapy

## Abstract

**Background:**

Neonatal jaundice affects most newborns worldwide and remains a major cause of preventable bilirubin-induced neurological injury in low-resource settings. Although most clinical guidelines recommend standardised electrically powered phototherapy and discourage sunlight exposure for newborns with jaundice due to safety concerns, uncontrolled exposure to direct sunlight remains widespread in many communities. We aimed to chart cross-cultural beliefs and practices regarding sunlight exposure for newborn jaundice to inform safer newborn care.

**Methods:**

We conducted a scoping review using the Arksey and O’Malley methodological framework and PRISMA-ScR guidance. PubMed, Scopus, Medline, Embase, Global Health, APA PsycINFO, Allied & Complementary Medicine, and other sources were searched for studies published between January 2000 and April 2026. Eligible studies included reports on knowledge, attitudes, practices, or recommendations regarding sunlight exposure for neonatal jaundice among mothers, caregivers, healthcare workers, or within health systems. Data were extracted using a standardised charting form and synthesised descriptively by geographical region. The review protocol was registered on the Open Science Framework (OSF).

**Results:**

Of 1,043 records, 109 studies from 30 countries met the inclusion criteria: Sub-Saharan Africa (*n* = 40), Middle East/North Africa (*n* = 25), South Asia (*n* = 22), East Asia/Pacific (*n* = 13), Europe (*n* = 3), North America (*n* = 3) and Latin America/Caribbean (*n* = 3); 88% were quantitative cross-sectional surveys of mothers and caregivers. Most of the included studies (87/109 or 79.8%) were identified outside the major databases. The proportion of respondents endorsing or practising unsupervised sunlight exposure ranged from 1.1 to 100% with marked regional gradients. Health-care workers were a major source of advice, with up to 84% of nurse-midwives recommending sunlight in some settings. Early-morning unfiltered exposure (08:00–10:00 h) for 15–30 min daily was the modal practice. Adverse effects, including sunburn, dehydration, hyperthermia and delayed facility presentation, were under-reported.

**Conclusion:**

Despite safety concerns and recommendations in several clinical guidelines in high-income countries discouraging direct sunlight exposure for neonatal jaundice, unsupervised sunlight exposure for newborn jaundice remains widespread, professionally endorsed, and inadequately scrutinised for potential harm. Improving access to conventional phototherapy, strengthening caregiver education, and evaluating safe alternatives in resource-constrained settings should be prioritised to ensure optimal newborn care.

## Introduction

Worldwide, some 60–80% of newborns develop jaundice before or after hospital discharge. Routine screening with a transcutaneous bilirubinometer is recommended to promptly identify newborns with or at risk of severe hyperbilirubinaemia, based on total serum or plasma bilirubin (TSB) levels (1, 2). The primary treatment is phototherapy and/or exchange transfusion. A standard phototherapy device is typically electric-powered and has a spectral irradiance of 8–10 μW/cm^2^/nm and a blue-green wavelength of approximately 478 nm (range: 460–490 nm), delivered to at least one surface of the body (ventral or dorsal) (1). Intensive phototherapy with a spectral irradiance of ≥30 μW/cm^2^/nm (range: 25–35 μW/cm^2^/nm) is recommended to effectively treat newborns with severe hyperbilirubinaemia.

Despite these recommendations, exposing newborns to direct sunlight without supervision to prevent or treat jaundice is not uncommon, especially in low-resource settings where electricity is unavailable or unreliable (3–9). For example, it is estimated that at least 15% of health facilities in sub-Saharan Africa and 12% in South Asia lack access to electricity, with even lower access in rural areas (10). Furthermore, only about half of the hospitals in sub-Saharan Africa have a reliable power supply. Although accidental exposure of a jaundiced baby to sunlight by a nurse in the United Kingdom in 1956 laid the foundation for the development of modern phototherapy (11, 12), most clinical guidelines for newborn care recommend routine screening for neonatal jaundice but prohibit the exposure of newborns to sunlight in any form because of safety concerns from potential hyperthermia, sunburn, dehydration, and skin cancer (1, 13). However, caregivers in low-resource settings face ethical challenges with these guidelines when they are presented with newborns at risk of severe hyperbilirubinaemia and bilirubin-induced mortality or neurological damage because of a lack of access to conventional phototherapy (14, 15). Moreover, most newborns develop severe jaundice at home after discharge from hospitals and birthing centres (15–18). Additionally, of the 132 million children born annually worldwide, one in five are delivered outside health facilities (19). Exposure to sunlight often becomes a default, less preferred but inevitable option in many settings, as part of the balancing act between the benefits and risks of treatment. Caregivers’ beliefs and practices, therefore, influence health-seeking behaviours and play a significant role in optimising newborn care to reduce infant morbidity and mortality across populations (2, 20, 21). To the best of our knowledge, no study has systematically mapped the evidence in the literature on the beliefs and practices associated with sunlight exposure in newborns, specifically regarding the prevention or treatment of jaundice worldwide. This scoping review set out to address this gap to support the need for public health initiatives to harness sunlight safely in settings where access to conventional phototherapy is constrained.

## Methods

The scoping review was conducted based on the methodological framework developed by Arksey and O’Malley (22), with improvements by Peters et al. (23), which required: (a) identifying a research question, (b) identifying relevant studies, (c) selecting the studies, (d) charting the data, and (e) collating, summarising, and reporting the results. The review also complied with the PRISMA Extension for Scoping Reviews (PRISMA-ScR) (24). The study protocol was registered on the Open Science Framework (OSF) (25).

### Identifying the research questions

Based on the Population–Concept–Context (PCC) framework (23), the primary research question was: How common is the belief and practice of exposing newborns to sunlight to treat jaundice worldwide? The population included neonates, infants, and caregivers (including mothers, grandmothers, parents, and healthcare providers). The concept was sunlight exposure in the neonatal period, including its use for the prevention or treatment of jaundice. The context included all geographical regions and healthcare settings. The secondary research question was: What specific practices are associated with sunlight exposure, such as time of day, duration, and direct versus indirect exposure?

### Identifying relevant studies

On 4 May 2026, we searched PubMed, Scopus and ProQuest [for Medline, Embase, Global Health, APA PsycINFO, Allied & Complementary Medicine (AMED)] from January 2000 to April 2026. The year 2000 was chosen to mark the start of the most consequential global child health initiative under the United Nations Millennium Development Goals, 2000–2015 (MDGs), which were later replaced by the Sustainable Development Goals, 2015–2030 (SDGs). The search terms were (newborn* OR neonat*) AND (sun OR sunlight OR heliotherapy) AND (jaundice OR hyperbilirubin*) to align with the population-concept-context components that entail defining a search string for each component and combining them into the final search string (Appendix 1). The search results through ProQuest were set to automatically exclude duplicates across the included databases. Additionally, based on our knowledge of traditional practices for newborn care in low- and middle-income countries, we conducted Google Scholar and HINARI searches to identify relevant articles from journals not indexed in traditional scientific databases. The results were supplemented by snowball sampling of references from relevant articles. These results were combined with other search results from the primary databases in an Excel sheet, and duplicates were manually removed by one reviewer (BOO) based on the study title.

### Study selection

The inclusion and exclusion criteria are summarised in Table 1. Three reviewers (EOA, OIO and BOO) independently screened titles, abstracts, and full texts, with disagreements resolved by consensus. We excluded the more recent clinical trials of filtered-sunlight phototherapy and related studies to establish the available evidence on the traditional practice of unsupervised or uncontrolled sunlight exposure for newborn jaundice. Data were charted using a standardised form that captured study characteristics and key findings on the use of sunlight exposure for jaundice. Given heterogeneity in study designs and outcomes, findings were synthesised descriptively and organised by region. As a scoping review, no meta-analysis or formal risk-of-bias assessment was undertaken, as these are not mandatory under PRISMA-ScR guidelines (22). Additionally, a critical appraisal of individual sources of evidence was not considered essential to the primary objective of mapping all published evidence on sunlight exposure for neonatal jaundice, regardless of the data source (23, 24).

**Table 1:** Inclusion and exclusion criteria for studies.

Subject	Inclusion criteria	Exclusion criteria
Dates	January 2000–April 2026	Before 2000
Language	English	Non-English
Country	All countries	None
Full text in English	Available	Not available
Study type	Observational studies including qualitative, quantitative or mixed methods. Empirical data presented.	Protocols, editorials, opinions, commentaries, letters. No design or methods presented. No empirical evidence presented
Population (P)	Newborns, parents, caregivers including health workers.	Experimental, *in vitro* studies
Intervention type (I)	Sunlight exposure or sunbaths for neonatal jaundice not supervised by health workers	Controlled or supervised sunlight exposure. Jaundice treatment without mentioning sunlight exposure, Sunlight exposure without mentioning neonatal jaundice. Primarily focused on vitamin D. Only mentioned exposure to (neon or day) light.
Context (C)	No restrictions. Community or hospital based	
Outcomes (O)	Intended or actual newborn exposure to sunlight to treat jaundice to reflect belief or practice.	

### Charting and summarising the data and reporting the results

A data charting form was developed on Microsoft Excel to extract the following information from the selected studies: country of study, publication title, first author, year of publication, study design, study settings, sample size, respondent type, and key findings on sunlight exposure for the treatment or intended treatment of neonatal jaundice by caregivers such as beliefs, practices, method, timing, and duration of exposure. Countries of study were organised by geographical regions and per capita income levels based on the World Bank classifications. These consisted of seven regions (East Asia and the Pacific, Europe and Central Asia, Latin America & the Caribbean, Middle East & North Africa, North America, South Asia and Sub-Saharan Africa) and four income groups (Low Income, Lower-Middle Income, Upper-Middle Income, and High Income). The intended use of sunlight exposure was classified as a belief and distinguished from any reported actual sunlight exposure of a newborn. Two reviewers (EOA and BOO) independently extracted these data and subsequently merged them after reaching agreement on the information considered relevant to this review, including beliefs and practices regarding sunlight exposure to prevent or treat newborn jaundice, as well as frequency among respondents in any survey. The inclusion of any study that did not fully meet the criteria for inclusion but provided valuable information relevant to the review, especially from countries with limited literature on sunlight exposure for neonatal jaundice, was determined by consensus. A third reviewer (OIO) was consulted as needed to resolve any disagreements. An open-source software, Python v3.9.6 (Python Software Foundation, PSF License) was used for figure-generation from the included studies.

## Results

### Overview of search results

**Figure 1:**
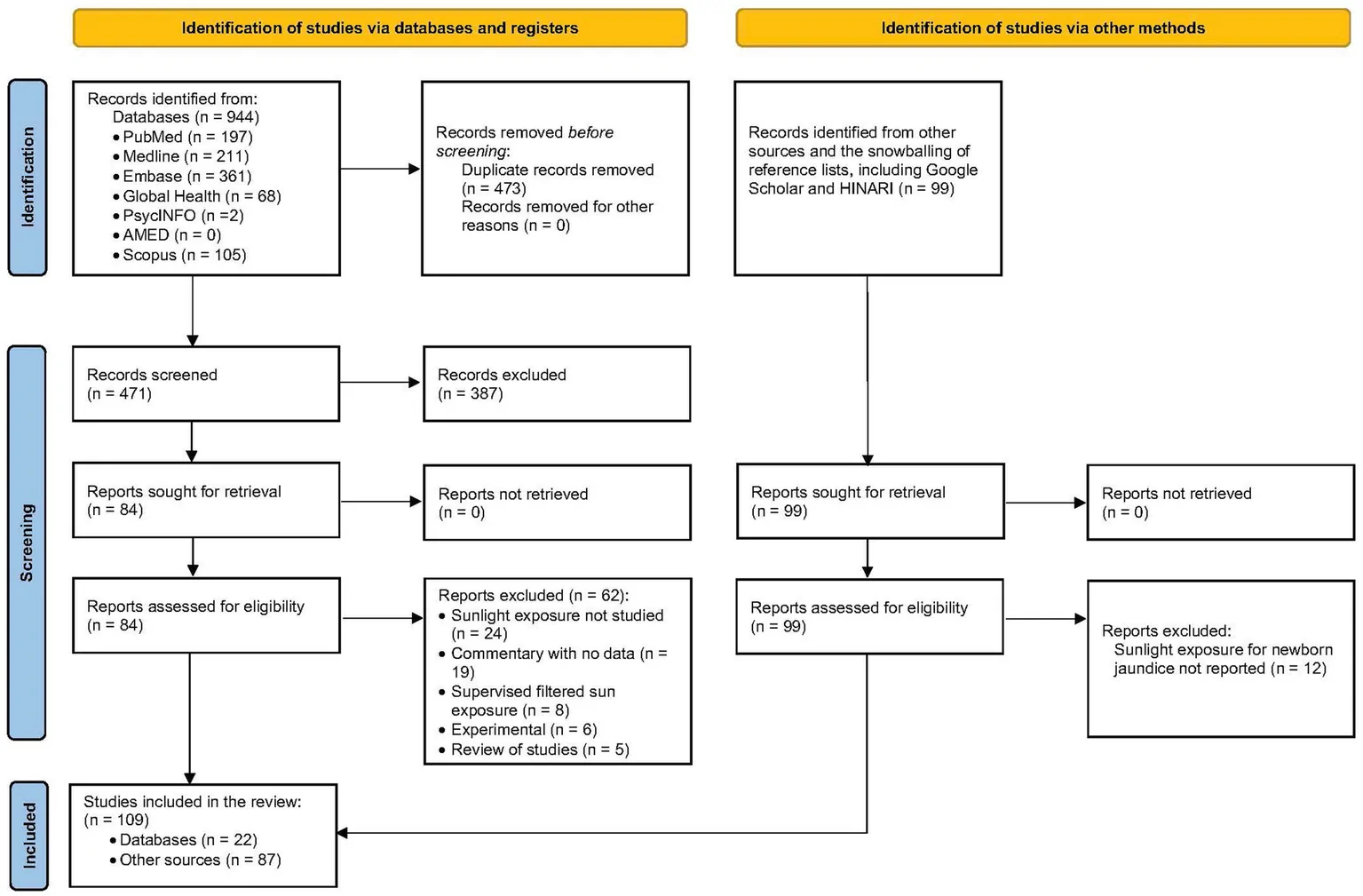
PRISMA flow diagram for study selection.

The selection process for the eligible studies is summarised in Figure 1. Database searches yielded 944 records, with an additional 99 articles from other sources, including a snowballing of references, for a total of 1,043 records. From the main databases, 473 duplicates were removed; the titles and abstracts of 471 articles were screened, and 387 were excluded for failing to meet the inclusion criteria. The full texts of the remaining 84 articles were reviewed, and 62 were excluded for various reasons, including lack of relevant information (*n* = 19), supervised filtered sun exposure (*n* = 8), experimental (*n* = 6), and review of studies (*n* = 5) (Appendix 2). From the 99 records obtained from other sources, 12 articles were excluded because they did not investigate sunlight exposure for the prevention or treatment of newborn jaundice. A total of 74 records were excluded across all sources for failing to meet the inclusion criteria, leaving 109 records that met the criteria (Appendix 3). Most of the included studies (87/109, 79.8%) were sourced outside traditional databases such as PubMed, Medline, Embase and Scopus.

### Characteristics of included studies

**Figure 2:**
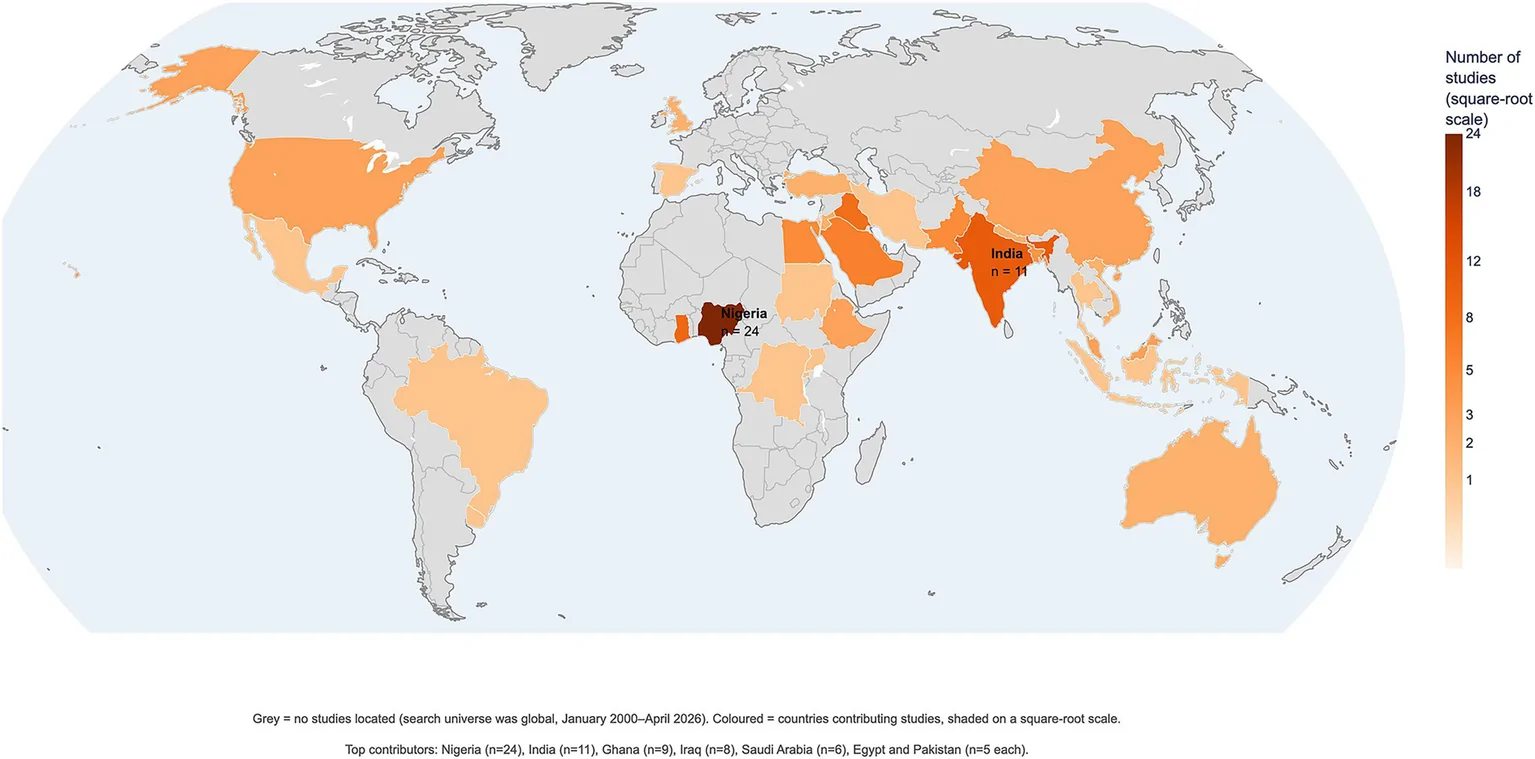
World map showing the distribution of included studies.

The key findings from the included studies are summarised in Table 2. The studies spanned all major world regions, with the largest contributions from Sub-Saharan Africa (40 articles), followed by the Middle East and North Africa (25 articles), South Asia (22 articles), East Asia & Pacific (13 articles), Latin America & the Caribbean (3 articles), North America (3 articles) and Europe & Central Asia (3 articles). The world map showing the geographical distribution of included studies is presented in Figure 2. Nigeria recorded the largest number of articles (n = 24), followed by India (*n* = 11). The distribution by World Bank income groups reflected the underlying epidemiology: 64 studies (58.7%) were conducted in lower-middle-income countries, 22 (20.2%) in upper-middle-income countries, 16 (14.7%) in high-income countries, and 7 (6.4%) in low-income countries. The methodological profile of the corpus was strongly weighted toward descriptive observational research. Ninety-six studies (88%) used a quantitative cross-sectional survey design, nine (8%) used a qualitative cross-sectional design, and the remaining four studies comprised one prospective observational survey, one prospective cross-sectional study, two mixed-methods or convergent designs, one narrative report, and one qualitative audit. One experimental study from Spain was intentionally included for its unique and relevant information on sunlight exposure through residential windows, which was reported as a common practice for treating neonatal jaundice in that country.

**Table 2:** Summary of key findings from a scoping review of 109 studies on sunlight exposure for newborn jaundice between 2000 and 2026.

Region/Country	Income group	Authors, Year	Study title	Study design	Setting	Sample size	Respondent type	Key beliefs	Key practices	Sunlight use (%)	Method/Timing/Duration
Sub-Saharan Africa											
Democratic Republic of Congo	Low-income	Fanello et al., 2023	Prevalence and risk factors of neonatal hyperbilirubinemia in a semi-rural area of the Democratic Republic of Congo: a cohort study.	Quantitative cross-sectional survey	Hospital-based	354	Mothers	11 (3.1%) would expose the newborn to sunlight.	Four women (1.1%) reported that they had a previous newborn with jaundice, and none received phototherapy (one sunlight exposure, one sweetened water, one oral traditional treatment, and one an unspecified drug).	3.1	Direct daily exposure for about 45 min early morning (8:00–8:45 a.m.) for 3 to 5 days at hospital and back home
Ethiopia	Low-income	Anmut et al., 2017.	Mother’s knowledge and practice about neonatal danger signs and associated factors in Wolkite Town, Gurage Zone, SNNPR, Ethiopia.	Quantitative cross-sectional survey	Community-based	355	Mothers who gave birth within 12 months prior to survey	Among 15 mothers responding regarding jaundice, six (40%) reported that sunlight exposure would be used as home treatment.			Direct exposure. Timing and duration not reported.
Ethiopia	Low-income	Ashebir et al., 2022.	Attitudes of mothers attending public hospitals in Addis Ababa, Ethiopia, to neonatal sunlight exposure: a cross-sectional study.	Quantitative cross-sectional survey	Hospital-based	420	Mothers with neonates and those attending follow-up and immunisation clinics	Some 239 (56.9%) had poor knowledge, and 306 (72.9%) had poor practice of sunlight exposure.			Direct exposure. Morning 8:00 to 10:00 for 30 to 60 min (per recommendation in study)
Ethiopia	Low-income	Mesele et al., 2023	Mothers’ health care seeking behaviour for neonatal danger sign in southern Ethiopia: Community based cross–sectional study.	Quantitative cross-sectional survey	Community-based	410	Mothers who gave birth in the previous 12 months		Of 231 neonates who experienced danger signs, 18 (7.8%) mothers gave home therapy including sunlight exposure for jaundice.	7.8	Direct exposure. Timing and duration not reported.
Ghana	Lower-middle income	Amegan-Aho et al., 2019	Neonatal Jaundice: awareness, perception and preventive practices in expectant mothers.	Quantitative cross-sectional survey	Hospital-based	175	Expectant mothers	Among 133 expectant mothers aware of neonatal jaundice, 11 (8.3%) will expose their babies to sunlight to treat jaundice.		8.3	Direct exposure. Timing and duration not reported.
Ghana	Lower-middle income	Asiedu & Edzeani, 2024.	Assessing the awareness and perception of neonatal jaundice among expectant mothers in Ghana.	Quantitative cross-sectional survey	Hospital-based	334	Expectant mothers		Some 197 (58.9%) reported use cultural practices, including sunlight exposure, for treatment of neonatal jaundice, include sunlight exposure.		Direct exposure. Timing and duration not reported.
Ghana	Lower-middle income	Boateng et al., 2024	Awareness and knowledge level of puerperal mothers on neonatal jaundice: a qualitative study in Northern Ghana.	Qualitative cross-sectional survey	Hospital-based	15	Puerperal mothers	One mother reported “………………, in the house they (older women) said I should put the baby into the early morning sun because it wasn’t much, so I did that for 2 days and realised it (NNJ) wasn’t going, that’s why I brought my baby to the hospital, and now it’s better.” Another mother reported: “I think jaundice can be treated at the hospital when you seek early medical care because in the house our mothers say we should put the babies in the early morning sun or we go for the “Darri” (combination of herbs) to clean the fever away but you people say is not good, so we should bring the baby to the hospital for treatment.”			Direct exposure to early morning sun. Duration not reported.
Ghana	Lower-middle income	Cudjoe et al., 2025	Knowledge, attitude and practice of puerperal and expectant mothers on neonatal jaundice in selected health facilities in Tamale metropolis, Ghana.	Quantitative cross-sectional survey	Hospital-based	229	Puerperal and expectant mothers	Some 130 (56.8%) respondents would expose babies to early morning sunlight to treat jaundice.			Direct exposure to early morning sun. Duration not reported.
Ghana	Lower-middle income	Donkor et al., 2023	Neonatal jaundice management: knowledge, attitude, and practice among nurses and midwives in the Northern Region, Ghana.	Quantitative cross-sectional survey	Hospital-based	202	Nurses and midwives	Some 55 (27.2%) respondents believed that physiological jaundice can be treated at home with early-morning sun.		27.2	Direct exposure to early morning sun. Duration not reported.
Ghana	Lower-middle income	Salia et al., 2021	Knowledge, attitudes and practices regarding neonatal jaundice among caregivers in a tertiary health facility in Ghana.	Quantitative cross-sectional survey	Hospital-based	202	Caregivers (mother or father) who sought postnatal services	Only 61 (30.2%) would avoid exposing their babies to early morning sunlight to treat jaundice.		69.8	Direct exposure to early morning sun. Duration not reported.
Ghana	Lower-middle income	Seneadza et al. 2022	Neonatal jaundice in Ghanaian children: assessing maternal knowledge, attitude, and perceptions	Quantitative cross-sectional survey	Hospital-based	504	Mothers attending antenatal and postnatal clinics	Some 47 (9.3%) respondents will expose their babies to sunlight to treat jaundice.		9.3	Direct exposure. Timing and duration not reported.
Ghana	Lower-middle income	Wolski et al., 2024.	Community health worker knowledge and perceptions of neonatal jaundice in Kumasi, Ghana.	Qualitative cross-sectional survey	Hospital-based	23	Community health workers	Some 15 (65.2%) respondents considered sunlight as the ideal home treatment for jaundice. “If I check and it’s not severe, and I think it’s mild, then I tell the mother to continue to give her breastmilk.” “Maybe after exposing the baby for about three to 4 days to the sun, when the sun rises, and continuous breastfeeding, every thirty minutes.”			Direct exposure to early morning sun for 3 to 4 days.
Ghana	Lower-middle income	Zakaria et al., 2025	Maternal knowledge, attitudes, and determinants of practices in neonatal jaundice care at Tamale Teaching Hospital.	Quantitative cross-sectional survey	Hospital-based	248	Mothers attending the pre- and postnatal clinics	Some 155 (62.5%) respondents will expose their babies to sunlight to treat jaundice.		62.5	Direct exposure. Timing and duration not reported.
Nigeria	Lower-middle income	Adamu et al., 2023	Neonatal jaundice: evaluating knowledge and practice among mothers attending immunisation clinics of secondary health care facilities of Sokoto Metropolis.	Quantitative cross-sectional survey	Hospital-based	335	Mothers attending immunisation clinics	Some 93 (27.8%) respondents would expose the baby to sunlight to treat jaundice.		27.8	Direct exposure. Timing and duration not reported.
Nigeria	Lower-middle income	Adebami 2025	Assessment of knowledge on causes and care of neonatal jaundice at the Nigerian primary and secondary health institutions.	Quantitative cross-sectional survey	Hospital-based	219	Health workers		Among 141 birth attendants at primary care facilities, 118 (83.7%) reported that they will always expose jaundiced babies to early morning sunlight, and 19 (13.5%) sometimes did so. Of 68 midwives in secondary health facilities, 47 (69.1%) will always expose jaundiced babies to early morning sunlight, and 15 (22.1%) did so sometimes.	83.7	Direct exposure to early morning sun. Duration not reported.
Nigeria	Lower-middle income	Adigun et al. 2018	Newborn care practices and knowledge of risk factors associated with neonatal mortality among postnatal mothers in Ibadan.	Quantitative cross-sectional survey	Hospital-based	206	Mothers attending postnatal clinics	Some 22 (10.7%) respondents would expose the baby to early morning sunlight to treat jaundice.		10.7	Direct exposure to early morning sun. Duration not reported.
Nigeria	Lower-middle income	Ajator et al., 2025	The use of unconventional methods in the treatment of neonatal jaundice among mothers who are traders in Nkwo Nnewi, Anambra State, Nigeria.	Quantitative cross-sectional survey	Community-based	240	Mothers who are market traders		Some 204 (85.4%) respondents exposed their babies to sunlight treat jaundice.	85.4	Direct exposure. Timing and duration not reported.
Nigeria	Lower-middle income	Barclay et al., 2022	Neonatal jaundice: knowledge and practices of healthcare providers and trainees in Southwest Nigeria.	Quantitative cross-sectional survey	Hospital-based	323	Health care providers and trainees	Among health workers, 9/33 (27.3%) physicians, 47/60 (78.3%) nurses, 91/108 (84.3%) nurse midwives, 9/41 (22.0%) traditional birth attendants, 23/32 (71.9%) medical students and 27/49 (55.1%) nursing students would expose newborns to sunlight to treat jaundice. Many, including 100% of the physicians and 81.3% of the nurse midwives, considered early morning as the appropriate time for sunlight phototherapy.		78.3 (nurses)/84.3 (nurse midwives)/27.3 (physicians)	Direct exposure to early morning sun. Duration not reported.
Nigeria	Lower-middle income	Chime et al., 2023	Caregivers’ perception of common neonatal illnesses and their management among rural dwellers in Enugu state, Nigeria: a qualitative study.	Qualitative cross-sectional survey	Community-based	48	Female caregivers (mothers, grandmothers, aunts) of under-fives in rural communities	“Children with yellowness of the eyes are kept under the early morning sun; preferably before 10 a.m. daily till the yellowness of eyes cleared.”			Direct exposure to early morning sun before 10 a.m. daily until jaundice clears
Nigeria	Lower-middle income	Egube et al., 2013	Neonatal jaundice and its management: Knowledge, attitude, and practice among expectant mothers attending antenatal clinic at University of Benin Teaching Hospital, Benin City, Nigeria.	Quantitative cross-sectional survey	Hospital-based	389	Expectant mothers attending antenatal clinic	Of 389 respondents, 235 (60.4%) believed that jaundice could be treated by exposure of the baby to sunlight. If their babies had jaundice, 9 (2.3%) of 378 expectant mothers would expose their babies to sunlight at home.	Of 55 mothers who had previous experience with jaundice, 7 (1.8%) exposed their babies to sunlight at home.	60.4	Direct exposure. Timing and duration not reported.
Nigeria	Lower-middle income	Ekwochi et al., 2015	Neonatal jaundice: perception and care seeking behaviour among mothers/ caregivers in a developing Setting.	Convergent mixed-method study	Hospital-based	421	Mothers/caregivers attending infant welfare clinic		Of the 57 babies who required treatment for jaundice, mothers reported that 32 (56.1%) were exposed to sunlight.	56.1	Direct exposure to early morning sun. Duration not reported.
Nigeria	Lower-middle income	Eneh et al., 2009	Perception of neonatal jaundice among women attending children out-patient and immunisation clinics of the UPTH, Port Harcourt.	Quantitative cross-sectional survey	Hospital-based	225	Mothers attending immunisation clinics	Some 114 (50.7%) respondents believed that exposure to sunlight is the main treatment for neonatal jaundice.		50.7	Direct exposure. Timing and duration not reported.
Nigeria	Lower-middle income	Esan et al., 2022	Traditional beliefs in the management and prevention of neonatal jaundice in Ado-Ekiti, Nigeria.	Quantitative cross-sectional survey	Hospital-based	190	Mothers attending antenatal clinic.	Some 57 (30%) respondents would expose their babies to sunlight to treat jaundice.		30	Direct exposure. Timing and duration not reported.
Nigeria	Lower-middle income	Ezeaka et al., 2014	Pattern and predictors of maternal care-seeking practices for severe neonatal jaundice in Nigeria: a multi-centre survey.	Quantitative cross-sectional survey	Hospital-based	431	Pregnant mothers attending antenatal clinics	Some 38 (8.8%) would expose baby to sunlight to treat jaundice and of 128 mothers with prior experience of an infant with jaundice 51 (18%) considered sunlight exposure as a possible treatment for jaundice.		8.8	Direct exposure. Timing and duration not reported.
Nigeria	Lower-middle income	Ezeaka et al., 2016	Mothers’ perception of neonatal jaundice in Lagos, Nigeria: An urgent need for greater awareness.	Quantitative cross-sectional survey	Hospital-based	325	Mothers attending antenatal clinic.	Some 87 (26.8%) considered sunlight exposure as treatment for neonatal jaundice.		26.8	Direct exposure. Timing and duration not reported.
Nigeria	Lower-middle income	Ezeogu et al., 2024	Jaundice and treatment options: knowledge, views and current practices among caregivers of children attending a teaching hospital in Owerri, Nigeria.	Quantitative cross-sectional survey	Hospital-based	318	Mothers attending immunisation clinics	Some 207 (65.1%) respondents will expose babies to sunlight to treat jaundice.		65.1	Direct exposure. Timing and duration not reported.
Nigeria	Lower-middle income	Goodman et al. et al., 2015	Neonatal jaundice: knowledge, attitude and practices of mothers in Mosan-Okunola community, Lagos, Nigeria.	Quantitative cross-sectional survey	Community-based	270	Women of reproductive age (15–49 years).	Some 156 (57.8%) respondents will expose the baby to sunlight to treat jaundice.		57.8	Direct exposure. Timing and duration not reported.
Nigeria	Lower-middle income	Iliyasu et al., 2020	Care-seeking behaviour for neonatal jaundice in rural northern Nigeria.	Quantitative cross-sectional survey	Community-based	361	Women of reproductive age (15–49 years) who delivered within 12 months prior to the study.	Some 23 (6.4%) respondents will expose babies to sunlight to treat jaundice.		6.4	Direct exposure. Timing and duration not reported.
Nigeria	Lower-middle income	Ogunlesi et al., 2015	Maternal knowledge and care-seeking behaviours for newborn jaundice in Sagamu, Southwest Nigeria.	Quantitative cross-sectional survey	Hospital-based	98	Mothers of neonates admitted into special care baby unit.		Of 30 infants with jaundice who received initial care at home, 6 (20.0%) were exposed to sunlight.	20	Direct exposure. Timing and duration not reported.
Nigeria	Lower-middle income	Okperi 2013	Neonatal jaundice and birth asphyxia as major causes of cerebral palsy in Nigeria: are doctors’ wrong beliefs and practices part of the problem?	Quantitative cross-sectional survey	Community-based	136	Doctors	Some 51 (37.5%) respondents will recommend exposing babies to sunlight to treat jaundice.			Direct exposure to early morning sun. Duration not reported.
Nigeria	Lower-middle income	Okposio et al., 2014	Evaluation of knowledge and perception of newborn jaundice among parturient mothers in a secondary health care centre in the Niger Delta region of Nigeria.	Quantitative cross-sectional survey	Hospital-based	156	Parturient mothers	Some 28 (17.9%) respondents will expose babies to sunlight to treat jaundice.			Direct exposure. Timing and duration not reported.
Nigeria	Lower-middle income	Olatunde et al., 2020	Neonatal jaundice: perception of pregnant women attending antenatal clinic at a tertiary hospital in Southwest, Nigeria.	Quantitative cross-sectional survey	Hospital-based	102	Expectant mothers attending antenatal clinic	Some 35 (34.9%) respondents will expose the baby to sunlight to treat jaundice.			Direct exposure. Timing and duration not reported.
Nigeria	Lower-middle income	Onyearugha et al., 2016	Neonatal jaundice: evaluating the knowledge and practice of expectant mothers in Aba, Nigeria	Quantitative cross-sectional survey	Hospital-based	300	Expectant mothers attending antenatal clinic	Some 192 (64%) respondents believe that jaundice can be treated by exposure to sunlight.			Direct exposure. Timing and duration not reported.
Nigeria	Lower-middle income	Orimadegun et al., 2017	Primary health workers’ knowledge and practices relating to neonatal jaundice in Ibadan, Nigeria.	Quantitative cross-sectional survey	Community-based	420	Community health workers	Some 280 (66.7%) respondents believe that jaundice can be treated by exposure to sunlight.			Direct exposure. Timing and duration not reported.
Nigeria	Lower-middle income	Owolabi et al., 2022	Perception of mothers towards neonatal jaundice in Ekiti State University Teaching Hospital, Ado-Ekiti, Ekiti State.	Quantitative cross-sectional survey	Hospital-based	115	Expectant mothers attending antenatal clinic	Some 99 (86.0%) respondents agreed that exposure to sunlight is a form of treatment for neonatal jaundice and 16 (14.0%) disagreed.			Direct exposure to early morning sun. Duration not reported.
Nigeria	Lower-middle income	Shehu et al., 2020	Analysis of mothers’ knowledge, beliefs and practice towards neonatal jaundice in Bingham University Teaching Hospital Jos, Plateau State, Nigeria.	Quantitative cross-sectional survey	Hospital-based	119	Mothers attending antenatal and postnatal clinics	Some 54 (45.4%) respondents will expose babies to sunlight to treat jaundice.		45.4	Direct exposure. Timing and duration not reported.
Nigeria	Lower-middle income	West et al., 2021	Neonatal jaundice: knowledge, attitude and practice among pregnant women attending the antenatal clinic of Rivers State University Teaching Hospital, Nigeria.	Quantitative cross-sectional survey	Hospital-based	149	Mothers attending antenatal clinic.		Of 25 (16.8%) respondents whose babies had jaundice, 10 did not go to the hospital, and 4 (40%) from this group exposed their babies to early morning sun.	40	Direct exposure to early morning sun. Duration not reported.
Rwanda	Low income	Sheth et al., 2021	Does provider access to technology improve health care? Evidence from a national distribution of phototherapy in Rwanda.	Quantitative cross-sectional survey	National survey of 46 major hospitals.	48	Healthcare providers/hospitals.		Before phototherapy scale-up, 17·5% of infants diagnosed with jaundice were treated with direct sunlight because conventional phototherapy was unavailable. Following intervention, sunlight use declined by 6·96 percentage points.	17.5	Direct exposure. Timing and duration not reported.
Sudan	Low income	Mohammed Ahmed et al., 2022	Mother’s knowledge and attitude regarding recognition of neonatal signs of danger.	Quantitative cross-sectional survey	Hospital-based	188	Mothers of newborns in participating hospitals	Some 82 (43.6%) respondents will expose babies to sunlight to treat jaundice.		43.6	Direct exposure. Timing and duration not reported.
Uganda	Low income	Nalubega et al., 2024	Community Perceptions of Neonatal Infection in Uganda.	Qualitative cross-sectional survey	Community-based	97	Community leaders and pregnant women	One pregnant woman shared: “When I went back home, and they told me that the baby had jaundice, they advised me to put the baby under the morning sunlight and in the evening [sunlight].” A community leader: “The only and the best treatment for jaundice is putting the baby under the sun.”			Direct exposure to early morning sun and evening sunset. Duration not reported.
South Asia											
Bangladesh	Lower-middle income	Afroze et al., 2022	Mothers’ knowledge on neonatal jaundice attending a Neonatal Intensive Care Unit of a Tertiary Care Hospital in Dhaka, Bangladesh.	Quantitative cross-sectional survey	Hospital-based	324	Mothers of neonates admitted into Neonatal Intensive Care Unit (NICU)	Among 138 mothers who believed that babies with jaundice required treatment, 90 (65%) considered sunlight exposure as the option.		65	Direct exposure. Timing and duration not reported.
Bangladesh	Lower-middle income	Huq et al., 2017	Knowledge regarding neonatal jaundice management among mothers: A descriptive study done in a tertiary-level hospital of Dhaka city.	Quantitative cross-sectional survey	Hospital-based	150	Mothers attending postnatal clinics	Some 135 (90.6%) respondents considered sunlight exposure as treatment for jaundice.		90.6	Direct exposure. Timing and duration not reported.
Bangladesh	Lower-middle income	Rahman et al., 2023	Feasibility and acceptability of home-based neonatal hyperbilirubinemia screening by community health workers using transcutaneous bilimeters in Bangladesh.	Qualitative cross-sectional survey	Community-based	278	Mothers, grandparents, Health workers	Of 278 mothers, about half reported that the only treatment for jaundice is “keeping the baby under sunlight.” A few mothers reported keeping the baby in the sunlight but did not know it was a treatment for jaundice. Putting babies under sunlight is the most practised treatment method by mothers and grandparents.			Direct exposure. Timing and duration not reported.
Bangladesh	Lower-middle income	Rasul et al., 2010	Outcome of neonatal hyperbilirubinemia in a tertiary care hospital in Bangladesh.	Prospective cross-sectional study	Hospital-based	426	Medical record		Some 137 (32.3%) of the newborns improved with sunbaths alone.	32.3	Exposure for 1–2 h in early morning and afternoon, through tinted glass
India	Lower-middle income	Aggarwal et al., 2017	Neonatal jaundice: knowledge, attitudes, beliefs, and practices of postnatal mothers in a tertiary care hospital in Uttarakhand, India.	Quantitative cross-sectional survey	Hospital-based	350		Some 80 (23%) considered exposure to sunlight a prominent preventive and treatment practice, especially among Hindu mothers.		23	Direct exposure. Timing and duration not reported.
India	Lower-middle income	Arumugam et al., 2023	Traditional newborn care practices in a tribal community of Tamilnadu, South India: A mixed methods study.	Mixed methods survey	Community-based	59	Mothers with infants residing in the study area for more than one year.	Some 40 (67.8%) respondents will expose their jaundiced newborns to sunlight.		67.8	Direct exposure. Timing and duration not reported.
India	Lower-middle income	Barathi & Surumi 2019	Assess the cultural practices on newborn care among mothers residing in Kayamkulam.	Quantitative cross-sectional survey	Community-based	60	Mothers of infants aged 1–6 months		All (100%) of the 60 mothers surveyed reported that they exposed their jaundiced babies to sunlight.	100	Direct exposure. Timing and duration not reported.
India	Lower-middle income	Gaur et al., 2024	Cultural practices and beliefs followed for newborn care in Santhal Pargana - A cross-sectional study.	Quantitative cross-sectional survey	Hospital-based	429	Mothers attending postnatal clinics	Some 424 (98.8%), respondents believed that a baby’s skin should be exposed to sunlight in case of jaundice.		98.8	Direct exposure. Timing and duration not reported.
India	Lower-middle income	Khound et al., 2021	Knowledge, attitude and practice of mothers regarding neonatal jaundice: a hospital-based observational Study.	Quantitative cross-sectional survey	Hospital-based	439	Mothers of newborns with jaundice	Some 101 (23%) respondents believed exposing babies to sunlight could prevent or treat jaundice.		23	Direct exposure. Timing and duration not reported.
India	Lower-middle income	Metta et al., 2024	A prospective observational exploration of maternal awareness of neonatal jaundice and risk assessment.	Quantitative cross-sectional survey	Hospital-based	61	Mothers	Some 27 (44.3%) respondents considered sunlight exposure as treatment option for neonatal jaundice.			Direct exposure. Timing and duration not reported.
India	Lower-middle income	Purani et al., 2015	Knowledge, awareness and practice of postnatal care among mothers.	Quantitative cross-sectional survey	Hospital-based	200	Mothers attending antenatal and postnatal clinics		Some 30 (1.5%) had exposed their babies to sunlight to treat jaundice.	1.5	Direct exposure. Timing and duration not reported.
India	Lower-middle income	Reshman & Sujatha 2014	Cultural practices and beliefs on newborn care among mothers in a selected hospital of Mangalore taluk,	Quantitative cross-sectional survey	Hospital-based	157	Mothers		Some 115 (73%) exposed the baby to sunlight when the baby’s skin turned yellow, and 10 (6%) dressed the baby in yellow clothes during jaundice.	73	Direct exposure. Timing and duration not reported.
India	Lower-middle income	Sasikala et al., 2017	A study on traditional beliefs and practices in newborn care among women attending UHTC, NMC, Nellore A. P.	Quantitative cross-sectional survey	Hospital-based	180	Mothers attending postnatal clinics		Some 143 (79.4%) respondents exposed the baby to sunlight when the newborn had jaundice.	79.4	Direct exposure. Timing and duration not reported.
India	Lower-middle income	Singh et al., 2025	Knowledge and awareness of mothers regarding neonatal jaundice.	Quantitative cross-sectional survey	Hospital-based	320	Mothers	Some 194 (60.6%) respondents believed that sunlight exposure has a role in preventing jaundice, either during pregnancy (108 or 33.8%) or exposure of babies to sunlight (86 or 26.9%).		26.9	Direct exposure. Timing and duration not reported.
India	Lower-middle income	Vernekar et al., 2021	A study on traditional beliefs and practices during the postpartum period among mothers at a district hospital in Goa.	Quantitative cross-sectional survey	Hospital-based	80	Mothers attending postnatal clinics		Some 72 (90%) respondents exposed their babies to warm sunlight when they had jaundice.	90	Direct exposure to warm sunlight. Timing and duration not reported.
Nepal	Lower-middle income	Dharel et al., 2016	Maternal perception about neonatal jaundice in Eastern Nepal: a qualitative study.	Qualitative cross-sectional survey	Hospital-based	32	Mothers of infants under 6 months with history of neonatal jaundice		Some 16 (50%) believed that exposure to sunlight would cure jaundice. “When my baby had jaundice, few days after birth, I applied pure mustard oil more frequently and kept under the sunlight.” “The ‘health worker’ repeatedly told me not to worry, but to keep the baby in morning sunlight. Jaundice increased the next day, and we took her to ‘faith healer’ who did some rituals to take jaundice away and applied something on her body. I strictly avoided the foods as per his advice. Jaundice gradually subsided over a week.”		Direct exposure to morning sunlight. Timing and duration not reported.
Nepal	Lower-middle income	Shrestha et al., 2019	Knowledge about neonatal jaundice among Nepalese mothers.	Quantitative cross-sectional survey	Community-based	177	Mothers with a child under 2 years old.	Some 135 (76%) believed that exposure to early morning sunlight is the main treatment for jaundice.		76	Direct exposure to early morning sun. Duration not reported.
Pakistan	Lower-middle income	Altaf et al., 2025	Bilirubin profiles and symptomatology in neonates: unravelling gender and location specific trends in Shaheed Benazirabad.	Quantitative cross-sectional study	Hospital-based	200	Parents of neonates (1–14 days) with clinical jaundice		Some 84 (42%) neonates were treated by exposure to sunlight.	42	Direct exposure. Timing and duration not reported.
Pakistan	Lower-middle income	Hassan et al., 2025	Neonatal hyperbilirubinemia investigating the relationship between risk factors and jaundice.	Quantitative cross-sectional survey	Hospital-based	210	Mothers of neonates aged 0–28 days.		Study protocol provides that babies with jaundice should be exposed to sunlight if mother declines phototherapy for three to five days in the hospital. Back home, mothers are advised to expose their infants to sunlight every day for around forty-five minutes in the early morning (often between 8:00 and 8:45 a.m.). Of 156 neonates with jaundice, 38 (24.4%) were exposed to sunlight.		Direct exposure to early morning sun for ~45 min daily (8:00–8:45 a.m.), for 3–5 days
Pakistan	Lower-middle income	Qumer et al., 2022	Knowledge, attitude and practices of women towards the neonatal jaundice in Pakistan	Quantitative cross-sectional survey	Community-based	107	Women	Some 97 (90.6%) considered sunlight exposure as treatment for jaundice.		90.6	Direct exposure to morning sunlight. Timing and duration not reported.
Pakistan	Lower-middle income	Saddozai et al., 2022	Knowledge, attitude, and practice of mothers regarding neonatal jaundice in the hospital of KPK, Pakistan.	Quantitative cross-sectional survey	Hospital-based	400	Mothers admitted to paediatric and neonatal wards within the first 72 h of their postpartum period.	Some 387 (96.8%) respondents considered sunlight exposure as treatment for jaundice.		96.8	Direct exposure. Timing and duration not reported.
Pakistan	Lower-middle income	Yaqub et al.,2016	To assess the knowledge of mothers regarding neonatal jaundice presenting to Rawal Institute of Health Sciences, Islamabad.	Quantitative cross-sectional survey	Hospital-based	200	Mothers presenting with jaundiced neonates within first 2 weeks of life	Some 105 (52.5%) respondents had inadequate knowledge of jaundice including misconceptions regarding sunlight exposure.			Direct exposure. Timing and duration not reported.
Middle East and North Africa											
Egypt	Lower-middle-income	Elsakka et al., 2023	Mothers’ Knowledge and Caring Practices for Neonatal Physiological Jaundice: a multisite cross-sectional study in El-Beheira Governorate, Egypt.	Quantitative cross-sectional survey	Hospital-based, multi-centre	300	Mothers who have an infant under 6 months old with a history for neonatal physiological jaundice.		74 (24.7%) respondents exposed their newborns to sunlight to treat jaundice.	24.7	Direct exposure. Timing and duration not reported.
Egypt	Lower-middle-income	Farouk et al., 2018	Mothers’ knowledge and attitude regarding neonatal hyperbilirubinemia.	Quantitative cross-sectional survey	Hospital-based	375	Postpartum mothers (primipara and multipara)	Some 78 (20.8%) respondents believed that sunlight is helpful in treating jaundice.		20.8	Direct exposure. Timing and duration not reported.
Egypt	Lower-middle-income	Moawad et al., 2016	Perceptions, practices, and traditional beliefs related to neonatal jaundice among Egyptian mothers. a cross-sectional descriptive study.	Quantitative cross-sectional survey	Hospital-based	400	Mothers who gave birth within 1 month before the study.	Some 147 (36.8%) respondents viewed sunlight exposure as beneficial for treating jaundice.		36.8	Direct exposure. Timing and duration not reported.
Egypt	Lower-middle-income	Mostafa et al. 2019	Knowledge of neonatal hyperbilirubinemia among primary health care physicians: a single-centre experience.	Quantitative cross-sectional survey	Hospital-based	419	Primary care physicians	Some 57 (13.6%) respondents recommended exposing babies to sunlight to mothers to treat jaundice.		13.6	Direct exposure. Timing and duration not reported.
Egypt	Lower-middle-income	Sabra et al., 2026	Assessment of mothers’ knowledge and practices regarding neonatal Jaundice.	Quantitative cross-sectional survey	Hospital-based	200	Mothers of newborns with jaundice	Some 100 (50%) respondents would expose their babies to sunlight to treat jaundice.		50	Direct exposure. Timing and duration not reported.
Iran	Upper-middle income	Rabiyeepoor et al., 2014	To study the knowledge and attitude of postnatal mothers on neonatal jaundice in Motahari Hospital, Iran	Quantitative cross-sectional survey	Hospital-based	200	Mothers	Some 152 (76%) respondents did not believe that exposing newborns to sunlight would increase the risk of dehydration or worsen the babies’ condition.		76	Direct exposure. Timing and duration not reported.
Iraq	Upper-middle income	Al-ezzi et al., 2022	Neonatal jaundice: knowledge, practice, and attitude among primigravida women.	Quantitative cross-sectional survey	Hospital-based	165	Women of reproductive age (18–25 years)	Some 11 (6.7%) respondents would expose their babies to sunlight to treat jaundice.		6.7	Direct exposure. Timing and duration not reported.
Iraq	Upper-middle income	Al-Hadeethi et al., 2026.	Knowledge of mothers regarding traditional methods of neonatal jaundice treatment.	Quantitative cross-sectional survey	Hospital-based	283	Mothers of newborns	Some 16 (5.5%) respondents would expose their babies to sunlight to treat jaundice.		5.5	Direct exposure. Timing and duration not reported.
Iraq	Upper-middle income	Al-Zamili & Saadoon, 2020	Knowledge, attitude and practice of mothers to neonatal jaundice.	Quantitative cross-sectional survey	Hospital-based	101	Women of reproductive age	Some 4 (4.0%) respondents would expose their babies to sunlight to treat jaundice.		4	Direct exposure. Timing and duration not reported.
Iraq	Upper-middle income	Hameed et al., 2019	Assessment of mothers’ knowledge, practices, and beliefs toward home management of neonatal jaundice in two paediatric teaching hospitals.	Quantitative cross-sectional survey	Hospital-based, multi-centre	200	Mothers of newborns with jaundice	Some 38 (19%) respondents would expose their babies to sunlight to treat jaundice.		19	Direct exposure. Timing and duration not reported.
Iraq	Upper-middle income	Karim 2016	Home practices of Kurdish mothers regarding management of jaundice among newborns in Erbil City.	Quantitative cross-sectional survey	Hospital-based, multi-centre	130	Mothers attending postnatal clinics	Some 58 (44.6%) respondents would expose babies to sunlight to treat jaundice.		44.6	Direct exposure. Timing and duration not reported.
Iraq	Upper-middle income	Mohammed Kassim et al., 2021	The traditional practices of mothers in caring of neonates affected by hyperbilirubinemia.	Quantitative cross-sectional survey	Hospital-based	100	Mothers	Some 47 (47%) respondents always exposed, and 40 (40%) sometimes exposed their infants to sunlight to treat jaundice.		47	Direct exposure. Timing and duration not reported.
Iraq	Upper-middle income	Mohsin et al., 2021	Knowledge and attitudes of mothers towards neonatal jaundice in Karbala Teaching Hospital for Children.	Quantitative cross-sectional survey	Hospital-based	164	Mothers of newborns with jaundice	Some 44 (26.4%) respondents would expose their babies to sunlight to treat jaundice.		26.4	Direct exposure. Timing and duration not reported.
Iraq	Upper-middle income	Thabit 2019	Knowledge regarding neonatal jaundice among a sample of mothers attending some Primary Health Care centres/Baghdad.	Quantitative cross-sectional survey	Hospital-based, multi-centre	265	Mothers	Some 122 (46%) respondents would expose their babies to sunlight to treat jaundice.		46	Direct exposure. Timing and duration not reported.
Israel	High income	Dotan et al., 2025	Paediatricians’ knowledge and perception of the care of the infant with jaundice.	Quantitative cross-sectional survey	Hospital-based	188	Community paediatricians/physicians	Some 45 (23.9%) respondents would recommend sun exposure to treat neonatal jaundice.		24	Direct exposure. Timing and duration not reported.
Jordan	Upper-middle income	Al-Sagarat et al., 2017	Traditional practices adopted by Jordanian mothers when caring for their infants in rural areas.	Qualitative cross-sectional survey	Community-based	30	Women delivered a baby previously or had a baby whose age is equal or greater than a year.	Most used traditional methods including sunlight exposure for their infants, who had jaundice, rather than seeking medical advice and treatment. One reported; “In order to treat jaundice I exposed my son to sun and a room light, dressed him a garlic necklace and gave him water sweetened with sugar.” Another mother said: “I exposed my jaundiced baby to sun light during the morning when it was hot.”			Direct exposure. Timing and duration not reported.
Jordan	Upper-middle income	Tailakh et al., 2026	Knowledge, practices, and attitudes of Jordanian parents regarding neonatal jaundice associated with ABO incompatibility: a cross-sectional study.	Quantitative cross-sectional survey	Community-based	536	Mothers		Some 436 (81.4%) respondents exposed their babies to sunlight when they showed signs of jaundice.	81.4	Direct exposure. Timing and duration not reported.
Saudi Arabia	High income	Alfaifi et al., 2023	Knowledge and attitudes of parents regarding neonatal jaundice in Bisha City, Saudi Arabia.	Quantitative cross-sectional online survey	Community-based	242	Parents	Some 118 (48.8%) perceived sunlight as beneficial for treating jaundice. While	Some 35 (14.5%) exposed their babies to sunlight to treat jaundice.	14.5	Direct exposure. Timing and duration not reported.
Saudi Arabia	High income	Alfouwais et al., 2018	Assessment of knowledge, attitude and practice of Saudi Parents towards neonatal jaundice (NNJ): A Cross-sectional Study.	Quantitative cross-sectional survey	Community-based	4,413	Parents	Some 2,906 (65.9%) respondents considered sunlight to be beneficial for treating jaundice.	Some 602 (13.6%) exposed their babies to sunlight to treat jaundice.	13.6	Direct exposure. Timing and duration not reported.
Saudi Arabia	High income	Elghamrawy et al., 2022	Knowledge, attitude, and practice of neonatal jaundice: a cross-sectional study in the adult population in Saudi Arabia.	Quantitative cross-sectional survey	Community-based	335	Parents	Some 123 (36.70%) respondents considered sunlight beneficial for treating jaundice in babies.	Some 61 (18.30%) exposed their jaundiced babies to sunlight.	18.3	Direct exposure. Timing and duration not reported.
Saudi Arabia	High income	Hanif et al., 2022	The assessment of awareness level among the parents regarding neonatal jaundice in the Arar city, KSA.	Quantitative cross-sectional survey	Community-based	385	Parents	Some 144 (37.4%) respondents considered sunlight exposure as an effective treatment for jaundice and 114 (29.6%) will expose their babies to sunlight to treat jaundice.		29.6	Direct exposure. Timing and duration not reported.
Saudi Arabia	High income	Mahmoud et al., 2024	The prevalence, practice and attitude toward neonatal jaundice among Saudi mothers.	Quantitative cross-sectional survey	Community-based	689	Mothers aged 18 years and above with at least one child	Some 403 (58.7%) believed that a lack of sun exposure could indeed constitute a risk factor for neonatal jaundice and 450 (65.6%) considered exposure to early morning sun as an effective home treatment.		65.6	Direct exposure to early morning sunlight. Timing and duration not reported.
Saudi Arabia	Lower-middle income	Mudhol et al., 2024	Attitude and practice regarding sunning of babies among mothers of selected communities of selected area.	Quantitative cross-sectional survey	Community-based	50	Mothers having a child.	Some 49 (98%) respondents believed sunlight was good for babies, and 38 (76%) believed in exposing babies to sunlight to treat or prevent jaundice.	Most mothers, 41 (82.0%) sunned their babies in a shade, 41 (82.0%) sunned their babies before 10 a.m. and/or after 4 p.m. but 31 (62.0%) exposed their babies for longer duration than 15 min.	82	62% exposed baby directly for >15 min; 82% sunned before 10 a.m./after 4 pm; 82% in shade.
Turkey	Upper-middle income	Aladag et al., 2006	Parents’ knowledge and behaviour concerning sunning their babies: a cross-sectional, descriptive study.	Quantitative cross-sectional survey	Community-based	118	Parents attending to governmental primary healthcare units for their children’s routine vaccination in a period of 1 month	Some 15 (12.7%) respondents claimed that sunlight was good for jaundice.	Majority 97 (82.2%) exposed their babies to the sun outdoors and 42 (35.6%) exposed their babies to the sun indoors in front of a window.	82.2	Direct exposure. 52% sunned before 10-11 a.m. and/or after 3 pm; 44.9% exposed >15 min
Turkey	Upper-middle income	Cakmak et al., 2009	Maternal beliefs and attitudes concerning neonatal jaundice in Southeast Turkey.	Quantitative cross-sectional survey	Hospital-based	224	Mothers of hospitalised infants (1–21 days) with NNJ	Sun exposure was the most well-known traditional remedy for jaundice among 188 (83.9%) respondents.	Of the 122 (54.5%) who declared that they had used traditional methods to treat jaundice, sun exposure was reported by 8 (6.5%) mothers.	6.5	Direct exposure. Timing and duration not reported.
East Asia and the Pacific											
Australia	High income	Harrison et al. 2002	An investigation of professional advice advocating therapeutic sun exposure.	Quantitative cross-sectional survey	Hospital-based	278	Healthcare workers (nurses and doctors/medical staff)	Among 278 nurses, 117 (42.1%) would recommend sunlight exposure to mothers to treat jaundice. From this group of nurses, 24 (20.5%) would suggest exposure through a window, and 78 (66.7%) would specify indirect or filtered sunlight. Only 20.5% of the nurses who were in favour of using sunlight to treat neonatal jaundice specified an exposure period (13 suggested ‘brief exposure’, eight recommended 10 min, two recommended 15 min, and one recommended 30 min), and relatively few (12.5%) suggested restricting periods of exposure to early morning or late afternoon.		49.5% nurses/34.9% doctors recommend sunlight for NNJ	Direct exposure. 13 suggested brief exposure; 8 recommended 10 min; 2 recommended 15 min; 1 recommended 30 min; only 12.5% restricted to early morning/late afternoon
Australia	High income	Harrison, Nikles et al., 2013	Do not burn the baby: Advice from Australian nurses recommending therapeutic sun exposure during infancy.	Quantitative cross-sectional survey	Hospital-based, multi- centre	635	Healthcare workers (midwives, nurses)	Study participants were 635 midwives, registered nurses in the Australian Capital Territory (ACT) or Queensland (QLD). Many recommend exposing infants to sunlight for suspected neonatal jaundice (QLD 41.9%, ACT 75%). Some suggested exposure either through a window (QLD 20.5%, ACT 38.6%) and/or indirect or filtered sunlight (QLD 47%, ACT 15.8%). However, relatively few specified a maximum exposure period (QLD 20.5%, ACT 29.8%); recommendations in QLD ranged from “brief exposure” to 30 min and in the ACT from a few minutes to 40 min per day. The average exposure period suggested through a window was 8.3 min in the ACT and 7.5 min in QLD. Where exposure through glass was not stipulated, the average exposure period suggested was 16 min in the ACT and 8.6 min in QLD. The prevalence of these recommendations was highest in the ACT (75%), followed by tropical QLD (45.8%) and sub- tropical QLD (38.2%).		49.6% QLD/75.3% ACT nurses endorse sunlight for NNJ	Direct and indirect exposure. Average suggested exposure 8.3 min (ACT)/7.5 min (QLD) through window; 16 min (ACT)/8.6 min (QLD) direct
China	Upper-middle income	Chen et al. 2006	Neurodevelopmental outcome of severe neonatal hemolytic hyperbilirubinemia.	Narrative report	Hospital-based	128	Hospital staff		Some children with jaundice from rural areas where there was no phototherapy were later diagnosed with cerebral palsy and hearing loss as the jaundice was left to follow its natural course. Some mothers were advised by health care workers to have their babies exposed to sunlight.		Direct exposure. Timing and duration not reported.
China	Upper-middle income	Liu 2008	Preventive effect of Chinese herbal medicine soaking bath plus sunbathing on neonatal jaundice.	Quantitative cross-sectional study	Hospital-based	960	Mothers of newborns with jaundice		Sunbathing method: in the morning and afternoon each day, in direct sunlight (with eyes covered by an eye mask; baby boys should protect their testicles) for 0.5 to 1 h, usually in a well-lit place.		Direct exposure for 0.5–1 h, morning and afternoon, (eyes covered, testicles protected)
China	Upper-middle income	Zhang et al., 2015	Prenatal training improves new mothers’ understanding of jaundice.	Quantitative cross-sectional survey	Hospital-based	767	First-time mothers		Some 61 (8.0%) respondent exposed their babies to sunlight to treat jaundice.	8	Direct exposure. Timing and duration not reported.
Indonesia	Upper-middle income	Sampurna et al., 2021	The knowledge of Indonesian paediatric residents on hyperbilirubinemia management.	Quantitative cross-sectional survey	Hospital-based	242	Paediatric residents	Some 114 (47.1%) respondents viewed sunlight exposure as an alternate treatment to phototherapy for neonatal hyperbilirubinemia.		47.1	Direct exposure. Timing and duration not reported.
Malaysia	Upper-middle income	Adeeb et al., 2016	Knowledge and attitude of neonatal jaundice – Orang Asli perspective.	Quantitative cross-sectional survey	Community-based	102	Adults (male and female) aged 18 years and above	Some 48 (47%) respondents would expose the baby to the sun to treat jaundice.		47	Direct exposure. Timing and duration not reported.
Malaysia	Upper-middle income	Boo et al., 2011	Malaysian mothers’ knowledge and practices on care of neonatal jaundice.	Quantitative cross-sectional survey	Hospital-based	400	Pregnant or postpartum mothers.		Of 154 mothers, 128 (83.1%) of the multiparous mothers with a past history of a child with jaundice, practised placing their infants under the direct sun.	83.1	Direct exposure. Timing and duration not reported.
Malaysia	Upper-middle income	Ng & Chong, 2014.	What do mothers know about neonatal jaundice? Knowledge, attitude and practice of mothers in Malaysia.	Quantitative cross-sectional survey	Hospital-based	198	Mothers of newborns with jaundice	Some 144 (72.7%) respondents believed that sunlight was effective in treating neonatal jaundice, but only 22 (11.2%) will place their babies under the sun.		11.2	Direct exposure. Timing and duration not reported.
Singapore	High income	Poon et al., 2007	Survey on parenting practices among Chinese in Singapore.	Quantitative cross-sectional survey	Hospital-based	93	Mothers of newborns	Some 47 (50.5%) respondents considered placing a child in sunlight was an acceptable method to prevent NNJ.		50.5	Direct exposure. Timing and duration not reported.
Thailand/Myanmar	Lower-middle income	Prins et al., 2017	A survey of practice and knowledge of	Quantitative cross-sectional survey	Hospital-based, multi- centre	522	Pregnant women	Some 73 (14.0%) respondents would expose their babies to sunlight to treat jaundice.		14	Direct exposure. Timing and duration not reported.
Vietnam	Lower-middle income	Dung et al., 2025	Mother’s practices on newborn care with jaundice at the Mountain General Hospital, Vietnam.	Quantitative cross-sectional survey	Hospital-based	280	Mothers of newborns		Some 106 (37.9%) respondents used sunlight exposure to detect jaundice.	37.9	Direct exposure. Timing and duration not reported.
Vietnam	Lower-middle income	Le et al., 2014	Care practices and traditional beliefs related to neonatal jaundice in northern Vietnam: a population-based, cross-sectional descriptive study.	Quantitative cross-sectional survey	Community-based	979	Mothers of newborns	Some 267 (27%) respondents believed that sunlight exposure was beneficial for treating jaundice.		27	Direct exposure. Timing and duration not reported.
Latin America and The Caribbean											
Brazil	Upper-middle income	Fajardo et al. 2025	Assessment of medical team knowledge on sun exposure and neonatal jaundice in a hospital in Southern Brazil.	Quantitative cross-sectional survey	Hospital-based	49	Healthcare workers (physicians/paediatricians caring for newborns)	Among 47 physicians, 24 (51.1%) believed that sun exposure should be daily, 17 (37.0%) believed that the best time for sunbathing was before 10 a.m. and after 4 p.m., 18 (38.3%) stated that the duration of exposure to sunlight should be 10 to 15 min.		51.1	Direct exposure. 22 (47.8%) recommended 10–15 min; 12 (26.1%) at least 10 min; 56.1% recommended before 10 a.m. and after 4 pm
Mexico	Upper-middle income	Jiménez-Díaz et al., 2024	Neonatal jaundice detection in low-resource Mexican settings: possibilities and barriers for innovation with mobile health.	Qualitative cross-sectional survey	Hospital-based	19	Healthcare workers (doctors, nurses, health administrators)	All mentioned unfiltered sun exposure for newborns as a preventive and/or treatment measure for jaundice.	Mothers were advised to expose their babies to sunlight if they observe signs of jaundice or to seek hospital care when necessary.		Direct exposure. Timing and duration not reported.
Uruguay	High income	Lores et al., 2025	Understanding the maternal perception of neonatal jaundice: Insights from a hospital in Uruguay.	Quantitative cross-sectional survey	Hospital-based	201	Postpartum mothers		Approximately 25.4% (51/201) mentioned sun exposure as treatment	25.4	Direct exposure. Timing and duration not reported.
North America											
USA	High income	Brethauer & Carey 2010	Maternal experience with neonatal jaundice.	Qualitative cross-sectional survey	Hospital-based	6	Mothers of newborns with jaundice		Among 6 mothers interviewed, 3 (50%) were advised to put their infants “in the sun” as treatment for hyperbilirubinemia. “With our first child, they said that at any time you can, get him in the sun…put him in the sun…strip him down…of course he was an October baby, so it was a little more difficult…we got the same information with this baby as well…the paediatrician that saw her at the hospital that was on call said that too…anytime that you can put her in the sunlight.”	50	Direct exposure. Timing and duration not reported.
USA	High income	Kuzniewicz et al., 2008	Risk factors for severe hyperbilirubinemia among infants with borderline bilirubin levels: a nested case–control Study.	Quantitative cross-sectional study	Hospital-based	310 neonates	Medical record		Some 46 (15%) were exposed to sunlight based on physician recommendation; however, this intervention did not appear to reduce the risk of severe hyperbilirubinemia (OR 1.30; 95% CI: 0.63–2.72).	15	Direct exposure. Timing and duration not reported.
USA	High income	Petrova et al., 2006	Management of neonatal hyperbilirubinemia: paediatricians’ practices and educational needs.	Quantitative cross-sectional study	Hospital-based	335	Paediatricians		Some 3 (1.1%) respondents recommended exposing babies to sunlight to treat jaundice.	1.1	Direct exposure. Timing and duration not reported.
Europe and Central Asia											
Spain	High income	Sequí-Canet 2026	Luz solar a través de los acristalamientos del domicilio y prevención de la ictericia neonatal [Sunlight through residential glazing and prevention of neonatal jaundice]	Narrative study	Hospital-based	NS	Not applicable (no human respondents; physical measurement study)	Professional advice to place the newborn “in the sun, behind the window” to prevent or treat jaundice is still common in Spain.			Indirect direct exposure. Timing and duration not reported.
UK	High income	Rennie et al., 2019	Learning from claims: hyperbilirubinaemia and kernicterus.	Qualitative/Audit	Hospital-based	20	Litigation case records of infants with kernicterus		8/20 (40%) of kernicterus cases used sunlight	40	Direct exposure. Timing and duration not reported.
UK	High income	Dann et al., 2026	Understanding community detection, testing and management of neonatal jaundice in term infants in the UK: a mixed methods study.	Quantitative cross-sectional survey.	Hospital-based	130	Midwives		UK midwifery leaders frequently suggested ‘watching and waiting’ in cases of suspected mild jaundice and sometimes recommended sunlight exposure.		Direct exposure. Timing and duration not reported.

Sample sizes varied across studies, with the largest being 4,413 mothers in one study from Saudi Arabia. Studies from multiple regions worldwide reported on the use, perceptions, or recommendations regarding sunlight exposure for neonatal jaundice. Sample sizes spanned three orders of magnitude, from six respondents in the smallest qualitative study to 4,413 respondents in the largest community-based survey (median 219, interquartile range approximately 130 to 420). The dominant respondent type was women in the perinatal period—postnatal mothers, expectant mothers attending antenatal clinics, and mothers of newborns with diagnosed jaundice—who featured as the primary respondent group in more than four-fifths of studies. A minority of studies sampled health-care providers (nurses, midwives, physicians, paediatricians, community health workers and traditional birth attendants), parents of both sexes, and paired mother–infant dyads.

**Figure 3:**
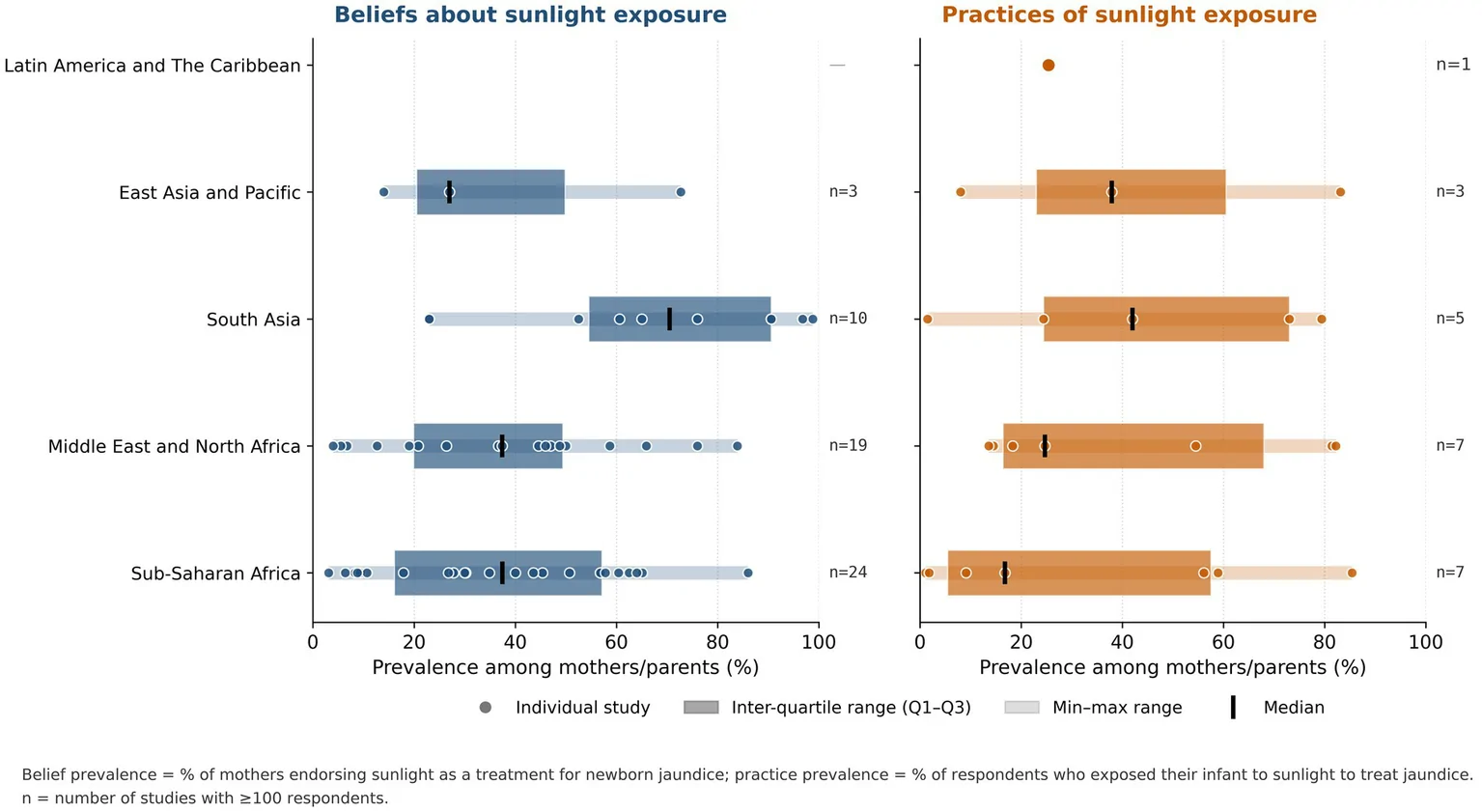
Prevalence of sunlight use among mothers.

The studies differed substantially in whether they assessed intended behaviour or beliefs regarding sunlight exposure or documented actual exposure among jaundiced infants. Many cross-sectional surveys evaluated maternal or caregiver intentions, beliefs, or perceptions about sunlight exposure. These studies typically asked whether mothers believed sunlight was an effective treatment or would expose their babies to sunlight if jaundice occurred. High proportions of intended use were reported in Ghana, India, Nepal, Nigeria, Pakistan and Saudi Arabia. By contrast, fewer studies documented actual exposure practices among jaundiced newborns. These studies reported that infants with recognised jaundice had already been exposed to sunlight at home or in healthcare settings. Overall, studies reporting actual exposure practices generally reflected resource limitations, delayed healthcare-seeking, or established traditional newborn-care behaviours, whereas studies assessing intentions often reflected cultural beliefs and the perceived effectiveness of sunlight therapy. The prevalence of sunlight use among caregivers (excluding health workers) in studies with at least 100 respondents is shown in Figure 3, separately for beliefs and practices. Beliefs about sunlight exposure were more commonly reported than actual practices in the included studies and may influence health-seeking behaviours that are not adequately captured in surveys. This gap is particularly noteworthy as the onset of severe jaundice predominantly occurs at home with the mothers, regardless of where the babies were born.

### Beliefs about sunlight exposure for neonatal jaundice

Key beliefs about sunlight exposure for neonatal jaundice were reported in 79 of the 109 studies (72%); in the remainder, the published account characterised practice without separately articulating the underlying belief. Across the 79 studies, the dominant belief—reported in every region represented in the corpus—was that exposure to natural sunlight, typically described in lay terms as “sunbathing” or “sunning” of the newborn, was an appropriate, available and effective treatment for neonatal jaundice or for the prevention of its progression. The proportion of respondents endorsing this belief varied widely between studies and within regions. In South Asia and parts of sub-Saharan Africa, single-study endorsement reached 100% (India and Mexico), with several studies from Ghana, India, Nigeria and Pakistan reporting endorsement above 80%. At the opposite extreme, several studies from Iraq, Saudi Arabia and high-income North American settings reported endorsement below 10%.

A second, less prevalent but recurrent belief, documented predominantly in qualitative studies from Bangladesh, Ghana, Iraq, Nigeria and Uganda, was that neonatal jaundice carried supernatural, ethno-aetiological or syncretic explanations—“bad blood,” “bad breast milk,” the evil eye, maternal dietary indiscretion, or environmental exposures during pregnancy—and that sunlight functioned as a culturally legitimate counter-measure that could be combined with herbal preparations, glucose water, or spiritual cleansing. A third recurrent belief, occasionally co-expressed with the first two, was the conflation of phototherapeutic effect with vitamin D acquisition or generic strengthening of the neonate. A fourth belief, distinctive to a small subset of studies from East and Southeast Asia (notably Vietnam, where confinement practices dictate active sunlight avoidance), was that sunlight exposure was either neutral or actively harmful to the newborn, and that the appropriate response to jaundice was darkness, warmth and indoor confinement.

Where studies sampled health-care workers directly, the endorsement of sunlight as a treatment for neonatal jaundice was substantial. In Australia, 42.1% of nurses in one survey and 49.6–75.3% in a later national survey of nurses across Queensland and the Australian Capital Territory endorsed the recommendation. In Brazil, 51.1% of paediatric providers believed that sunlight exposure should be daily, with 37.0% identifying the optimal exposure window as before 10:00 h or after 16:00 h. In Nigeria, 78.3% of nurses and 84.3% of nurse-midwives recommended sunlight, against 27.3% of physicians. In Spain, a recent paediatric review (Table 2) found that the professional recommendation to place the newborn “in the sun, behind the window” remained common. In the United States, the prevalence of provider endorsement was lower (1.1–18%), but a case–control study of physician advice to mothers of infants requiring hospital readmission for hyperbilirubinaemia found that recommended sunlight exposure was nominated by a small but non-negligible proportion of the index group.

### Practices of sunlight exposure for neonatal jaundice

Key practices were explicitly reported in 39 of the 109 studies (36%), and a quantitative estimate of the proportion of respondents who actually placed or recommended placing a jaundiced or at-risk neonate in natural sunlight was available from 83 studies. Across these 83 studies, the proportion ranged from 1.1% in the USA to 100% in India and Mexico, with a median of 40.0% (interquartile range 18.3–65.1%) (Table 2). Strong regional gradients were observed. The median prevalence of reported sunlight use was highest in South Asia at 73.0% (*n* = 17 studies; range 1.5–100%); intermediate in East Asia and the Pacific at 47.0% (*n* = 11; 8.0–83.1%) and Sub-Saharan Africa at 40.0% (*n* = 25; 3.1–85.4%); lower in the Middle East and North Africa at 26.4% (*n* = 24; 4.0–82.2%); and lowest in North America at 15.0% (*n* = 3; 1.1–50.0%). The single Latin American and Caribbean prevalence, with two extractable values, had a median of 51.1% (Brazil and Uruguay); a single European value of 40.0% was reported (United Kingdom kernicterus case series, in which 8 of 20 cases used sunlight).

In the studies where the practice was described in detail, three modal patterns recurred. The first was unfiltered direct exposure: the naked or partially clothed neonate placed on a balcony, veranda, courtyard or rooftop, with eyes uncovered and without explicit thermoregulatory or hydration safeguards; this pattern dominated lay practice in studies from South Asia (Bangladesh, India, Nepal, Pakistan), sub-Saharan Africa (Ethiopia, Ghana, Nigeria, Sudan, Uganda) and parts of the Middle East (Egypt, Iraq, Saudi Arabia). The second pattern was filtered or window-mediated exposure: the neonate placed indoors next to a closed window, on the explicit assumption that the glass attenuated harmful spectra while permitting therapeutic ones; this pattern was reported by minorities of providers in Australia (20.5% of pro-sunlight nurses suggested exposure through a window), the United States, parts of East Asia, and—notably—as the standard professional recommendation in the Spanish review. The third pattern, present in a minority of qualitative studies from Vietnam, Nepal and rural East Asia, was active avoidance, with the neonate kept in a darkened room during the confinement period.

### Sources of maternal information and advice

Mothers and caregivers commonly obtained information regarding sunlight exposure for neonatal jaundice from family members, older women, community traditions, and cultural practices. Qualitative studies from Ghana, Jordan, Nepal, Nigeria, and Uganda reported strong intergenerational influence, with grandmothers, older adults, traditional healers, and community leaders frequently advising mothers to expose infants to early-morning sunlight. Healthcare workers were also important sources of advice in several settings. Studies from Australia, Brazil, China, Mexico, Nigeria, Rwanda, and the United Kingdom reported that nurses, midwives, physicians, community health workers, and traditional birth attendants sometimes recommended sunlight exposure, particularly where access to phototherapy was limited.

### Direct versus indirect exposure

Most studies described the use of direct sunlight exposure, particularly in community and home settings across sub-Saharan Africa, South Asia, and the Middle East. Direct exposure to sunlight was implied where indirect exposure was not explicitly reported. Mothers commonly reported placing infants outdoors in early-morning sunlight or exposing unclothed infants directly to sunlight to treat jaundice. Several studies also described indirect or filtered exposure practices, especially in higher-resource settings. Australian nurses frequently recommended indirect sunlight or exposure to sunlight through glass windows. In Bangladesh, tinted glass filters were reportedly used to reduce harmful radiation from sunlight. Reports from Spain cautioned that residential windows still transmit substantial amounts of ultraviolet and infrared radiation despite their filtering effects. Sunlight exposure was commonly practised outside the house, especially in rural and low-resource settings, where infants were placed outdoors during early morning hours. Outdoor exposure was widely reported in studies from Bangladesh, Ghana, India, Pakistan, Nigeria and Uganda. Some studies also described exposure inside the house near windows or doorways. In Australia, nurses recommended exposing babies to the outdoors through windows or glass. In the United Kingdom, medico-legal reports documented advice from midwives suggesting that placing babies “in the window” would be sufficient treatment for jaundice. Turkish mothers similarly reported exposing babies indoors in front of windows.

### Time, duration and frequency of exposure

The early morning period was the most frequently recommended timing for sunlight exposure. Across studies from Ethiopia, Ghana, India, Nigeria, Pakistan, Saudi Arabia, and Uganda, caregivers and healthcare workers typically advised exposure between 08:00 and 10:00 h or before 10 a.m. Some studies also recommended late-afternoon or evening exposure to avoid intense midday sunlight. In Uganda and Bangladesh, participants described using sunlight at sunrise, sunset, or in the late afternoon. Australian nurses also frequently advised restricting exposure to early morning or late afternoon. Reported exposure duration varied considerably across settings. Some healthcare workers recommended only brief exposure, whereas others suggested prolonged daily exposure. Commonly reported durations ranged from 10–15 min to 30–60 min daily. Several studies recommended longer exposure periods. In Bangladesh and China, newborns were reportedly exposed for 0·5–2 h daily. In Pakistan and the Democratic Republic of Congo, mothers declining phototherapy were advised to expose infants for approximately 45 min daily over several days. Exposure was generally described as a daily practice until jaundice resolved. Some studies specified exposure over 3–5 consecutive days, particularly when phototherapy was unavailable or declined. In qualitative studies from Nigeria and Uganda, caregivers reported repeated daily morning exposures until the yellow discolouration cleared. In Australia and Brazil, some healthcare providers also recommended routine daily “sunbathing” for jaundiced infants.

### Awareness of potential harms and mitigants

Awareness of the potential harms associated with sunlight exposure was generally limited in many low-resource settings. Several studies showed that mothers and caregivers believed sunlight was harmless or beneficial, often associating it with the treatment of jaundice, the prevention of vitamin D deficiency, or general newborn wellbeing. However, some studies documented growing awareness of risks such as dehydration, overheating, delayed treatment, and severe hyperbilirubinaemia. In Iran, most mothers did not believe that sunlight could worsen neonatal conditions or increase the risk of dehydration. Conversely, reports from Spain, the United Kingdom, and the United States emphasised concerns regarding uncontrolled ultraviolet exposure, hyperthermia, sunburn, delayed access to phototherapy, and kernicterus. Only a minority of studies explicitly described measures intended to reduce ultraviolet injury or overheating. Reported protective strategies included limiting exposure to early morning or late afternoon periods; using indirect sunlight or sunlight through windows; placing infants in shaded areas; using tinted glass filters; and covering sensitive body parts. One Chinese study recommended covering infants’ eyes with masks and protecting male genitalia from direct sunlight. Saudi Arabian mothers frequently reported sunning infants in shaded areas and avoiding midday exposure. Despite these precautions, several studies and guideline-based reports have highlighted concerns about uncontrolled ultraviolet exposure, dehydration, hyperthermia, delayed treatment, and the risk of kernicterus associated with sunlight exposure.

## Discussion

This scoping review demonstrates that sunlight exposure remains a widespread practice for the prevention and treatment of neonatal jaundice globally, particularly in low- and middle-income countries. The findings reveal substantial heterogeneity in prevalence across and within geographical regions, with the highest estimates reported in South Asia and sub-Saharan Africa, and comparatively lower estimates in North America and Europe. These findings extend previous regional reviews from Nigeria and Ethiopia, showing that sunlight exposure remains deeply embedded in newborn-care traditions (6, 9), despite several clinical guidelines in high-income countries discouraging its use (1, 13), while those in LMICs rarely contain explicit recommendations regarding sunlight exposure (2, 26–28). In fact, the American Academy of Paediatrics (AAP) has clarified that its guidelines on neonatal jaundice, often regarded as the gold standard worldwide, are not intended for LMICs (1).

The consistently high prevalence reported in South Asia, particularly in Pakistan, Bangladesh, Nepal, and parts of India, suggests that sunlight exposure remains culturally normalised as a primary treatment for neonatal jaundice. In several studies, more than 90% of mothers believed sunlight was therapeutic. Similar observations have been reported in studies examining traditional neonatal practices in South Asia, where newborn care is strongly shaped by intergenerational advice, religious beliefs, and home-based postpartum customs (29–31). Traditional practices for newborns with jaundice are not limited to sunlight exposure. Invasive and non-invasive practices, such as cutting the baby’s heel, forehead, or under the tongue with a razor, puncturing the baby’s earlobe, traditional, herbal, and over-the-counter medication, glucose, sugar water or honey, mustard oil massage and body rubbing, religious rites, and dietary regulation for mothers are widely reported (32–35). The persistence of these practices may also reflect inadequate access to bilirubin screening, delayed postnatal follow-up, and limited awareness regarding severe hyperbilirubinaemia and kernicterus. Previous studies have shown that poor maternal knowledge of jaundice is associated with delayed healthcare seeking and increased risk of bilirubin-induced neurological injury (36).

Sub-Saharan Africa also demonstrated high and highly variable prevalence, particularly in Nigeria and Ghana. The wide range observed within countries probably reflects differences in study populations, urban–rural disparities, educational status, healthcare access, and local sociocultural beliefs. In Nigeria, prevalence ranged from approximately 6% to more than 85%, indicating that sunlight exposure is neither uniformly practised nor uniformly rejected. Importantly, several studies reported that some healthcare workers, including nurses, midwives, and physicians, endorsed sunlight exposure (37–40). This observation is consistent with reports from Australia, Israel, the United Kingdom, and the United States, showing that healthcare provider recommendations have historically contributed to the persistence of sunlight therapy despite formal guideline restrictions (41–44).

The review also highlights how structural limitations within health systems influence newborn-care behaviours. In many low-resource settings, sunlight exposure serves as a pragmatic substitute when conventional phototherapy is inaccessible, unaffordable, or unreliable. Studies from the Democratic Republic of Congo and Rwanda demonstrated that sunlight was used when phototherapy was unavailable or ineffective (45, 46). These findings align with broader evidence documenting inadequate electricity supply and limited neonatal-care infrastructure across many low-income countries (10, 47). Reliable phototherapy remains unavailable in many district hospitals and primary care facilities, particularly in rural areas. Moreover, sub-optimal and ineffective phototherapy devices prolong hospital stay and place a child at risk of adverse neurodevelopmental outcomes (48). Consequently, recommendations that categorically prohibit sunlight exposure may be difficult to implement where no feasible alternatives exist.

An important finding of this review was the substantial variation in the way sunlight exposure was practised. Most studies reported direct outdoor exposure during early morning hours, typically between 8:00 a.m. and 10:00 a.m., whereas others described indirect sunlight through windows, shaded exposure, or the use of tinted glass filters. Exposure duration ranged from a few minutes to several hours daily. This heterogeneity reflects the absence of standardisation and likely contributes to inconsistent therapeutic effects and unpredictable safety risks. Early-morning exposure may suffice for newborns with mild physiological jaundice, a fact that may have inadvertently encouraged this practice. However, avoiding exposure in the afternoon (12 noon to 4:00 p.m.), when irradiance is optimal, is likely to prolong treatment and increase the risk of severe hyperbilirubinemia for babies with pathological jaundice, particularly when associated with hemolytic conditions such as glucose-6-phosphate dehydrogenase (G6PD) deficiency and blood group incompatibility (49). Previous reports of kernicterus-related litigation in the United Kingdom, as well as case reports of neonatal sunburn, reinforce these concerns (43, 50).

The distinction between studies reporting maternal intention to expose infants and studies documenting actual exposure practices is also noteworthy. Many surveys assessed beliefs and intended behaviour rather than observed management of jaundiced infants. Although these findings demonstrate cultural acceptance of sunlight exposure, they may overestimate actual practice. Conversely, studies reporting actual exposure of jaundiced infants provide stronger evidence that sunlight is still actively used as a treatment in both home and clinical settings. These studies also suggest that sunlight exposure may delay timely presentation for medical evaluation, especially when jaundice is initially perceived as mild or self-limiting.

Among the suggestive but uncertain findings, the most important is the under-reporting of harm. Where the evidence describes practice in granular terms—the naked or partially clothed neonate placed in early-morning sunlight on a balcony or in a courtyard, with no explicit thermoregulatory or hydration safeguard—the inferred harm profile is non-trivial. Yet only a minority of studies in this review explicitly enquired about adverse effects, and where harms were recorded, they were typically described in terms of skin reddening or maternal discomfort rather than the bilirubin trajectory or facility-presentation timing that would establish a causal link to severe outcomes. The United Kingdom kernicterus case series (43), in which sunlight use co-occurred with delayed bilirubin testing in 40% of index cases, is the closest the corpus comes to a documented causal pathway; a similar pattern is suggested in qualitative reports from Iraq and Nigeria, in which mothers who used sunlight at home presented later to facility care than those who did not. The implication is that the harm signal is not absent from the literature but is systematically under-investigated, and that registry-level surveillance of sunlight-related adverse events would be a tractable research priority. The restriction of exposure to the suboptimal early-morning sun would suggest a minimal risk of harm from sunburn or dehydration. For example, in some countries such as Ethiopia, it is considered good practice by healthcare professionals for mothers to expose unclothed neonates to sunlight from 2 weeks of age for 15 to 30 min, twice or thrice a week, before 10:00 and after 16:00, to prevent vitamin D deficiency (6, 51, 52).

Despite these concerns, the review also provides important context for the emerging evidence on filtered-sunlight phototherapy. Experimental and clinical studies have shown that filtered sunlight systems can safely reduce ultraviolet and infrared exposure while retaining therapeutically effective blue-light wavelengths (53–57). The moderate risk of hyperthermia reported in the earlier trials (58) can be effectively controlled through close monitoring by trained health workers (56, 57). In settings where conventional phototherapy is unavailable, filtered sunlight phototherapy may represent a practical interim strategy that aligns more closely with existing community practices while improving safety and treatment monitoring. The persistence of sunlight exposure, as documented in this review and in other reviews (59), therefore underscores the need for context-sensitive approaches that acknowledge prevailing cultural practices while strengthening evidence-based neonatal care. Additionally, the evidence in this review and other studies (1, 53–57) refutes any erroneous suggestion or claim that sunlight therapy (with or without filters or amplifications) has no significant beneficial effect on infants with confirmed hyperbilirubinaemia (60).

This review has several strengths. To our knowledge, it is among the most comprehensive syntheses of global sunlight exposure practices for neonatal jaundice, encompassing 109 studies across diverse geographical regions and healthcare settings. The inclusion of studies from both indexed and non-indexed sources improved representation from low-resource settings where many traditional newborn-care practices are underreported in mainstream databases. The review also provides a detailed description of how sunlight exposure is practised, including timing, duration, location, and perceived protective measures.

### Limitations

This review has several limitations. First, the evidence presented is overwhelmingly cross-sectional and survey-based, with attendant risks of recall, social desirability, and selection bias in respondent recruitment. The included studies should be regarded as illustrative rather than representative of the countries. Second, the heterogeneity of prevalence definitions—whether respondents endorsed sunlight as a perceived treatment, used it in response to a current jaundice episode, or used it routinely as a preventive measure—limits direct numerical comparison across studies, and our summary statistics should be interpreted as descriptive rather than as pooled estimates. Third, studies that measured knowledge applied a wide range of scoring instruments, few of which were validated against a common standard, and our broad categorisation of knowledge as poor, moderate or good necessarily simplifies the underlying detail. Fourth, the corpus is unevenly distributed: 24 of the 109 studies were from Nigeria alone, while large populations in francophone Africa, South-East Asia outside Vietnam and Indonesia, Latin America outside Brazil and Uruguay, and Central Asia were minimally represented. Fifth, by limiting the inclusion criteria to English-language publications, relevant studies from Francophone sub-Saharan Africa and Spanish-speaking Latin America may have been systematically excluded, potentially distorting regional prevalence estimates. Sixth, our scoping review, by design, synthesises descriptive practice and belief data and does not formally evaluate the quality of the underlying studies; a subsequent systematic review with a formal risk-of-bias assessment would be warranted, particularly for the small experimental sub-literature on effectiveness. Finally, this review extends to publications up to early 2026; the very recent appearance of high-prevalence reports from settings such as Jordan and Ghana suggests that the literature is actively growing and that periodic updates will be required.

### Implications for practice, policy and research

The implications for practice are immediate. Clinicians, midwives and community health workers in every region represented in this review require explicit retraining that addresses unsupervised sunlight exposure not as a benign adjunct but as a potentially harmful substitute for guideline-recommended phototherapy, which should not be used to treat established neonatal hyperbilirubinaemia, if possible. National professional societies should issue unambiguous guidance to this effect, and undergraduate, postgraduate, professional, and continuing professional development curricula should be audited for any residual endorsement of the practice. Antenatal and postnatal patient-facing materials should be updated to address sunlight directly and to provide an evidence-based alternative—principally, early recognition of jaundice and timely referral to phototherapy-capable facilities where available.

The implications for policy are equally clear. In low- and middle-income countries, displacing sunlight as the modal home-management strategy is contingent on the provision of low-cost, locally maintained phototherapy. WHO and similar recommendations for optimal and essential newborn care should be updated to reflect the practical realities of the treatment options for infants with, or at risk of, severe jaundice outside hospital settings (2). In high-income settings, where access is not the limiting factor, the priority is provider behaviour change and, in jurisdictions where litigation data implicate sunlight in adverse outcomes, the inclusion of the practice in routine adverse-event review.

The implications for research are the most extensive. First, a registry-level study of adverse events associated with neonatal sunlight exposure is necessary to quantify, for the first time, the rates of sunburn, dehydration, hyperthermia and delayed-presentation kernicterus in real-world populations. Second, the persistence of professional endorsement in high-income settings—Australia, Brazil, the United States and Spain—deserves a dedicated qualitative investigation to identify the educational and cultural mechanisms by which an evidence-discordant recommendation survives. Third, the under-representation of francophone Africa, Central Asia and Latin America in the descriptive literature should be addressed by region-specific surveys to enable the targeting of interventions to local belief structures and information ecologies. Fourth, the contrast between high-prevalence sunlight-use cultures and low-prevalence sunlight-avoidance cultures within the same broad region deserves comparative ethnographic study; the cultural meanings ascribed to the postpartum infant and to natural light are clearly central to caregivers’ behaviour and have been examined in only a handful of qualitative studies in this review. Finally, community-oriented technologies that can safely harness the therapeutic benefits of sunlight for the treatment of neonatal jaundice, including solar-powered phototherapy, should be prioritised in global developmental assistance for childcare in low- and middle-income countries (49).

## Conclusion

Across 109 studies and almost a quarter of a century of literature, sunlight exposure for neonatal jaundice emerges from this scoping review as a globally prevalent, professionally endorsed and inadequately interrogated practice whose persistence is sustained as much by the clinical workforce as by the lay community. The belief base is rooted in plausibility rather than evidence; the practice base is heterogeneous in dose and timing; and the harm signal is suggestive but systematically under-measured. A coordinated programme of professional re-education, expansion of access to phototherapy, intergenerational community engagement, and registry-level harm surveillance is required to reduce the global burden of preventable bilirubin-induced neurological injury. Emerging but limited evidence from close monitoring of filtered-sunlight phototherapy suggests that a safe and effective alternative to direct sunlight exposure and other traditional, potentially harmful practices exists in settings where conventional electric phototherapy is unavailable or ineffective.
