# Supplementary material for: Cross-cultural beliefs and practices of caregivers regarding sunlight exposure for newborn jaundice: a scoping review
Source: Front Public Health. 2026 Aug 19;14:1888701. doi: 10.3389/fpubh.2026.1888701 (PMC13533943; doi:10.3389/fpubh.2026.1888701)
Supplement: Supplementary file 1 [file Data_Sheet_1.PDF]

Appendix 1. Search string for PubMed, adapted for ProQuest and Scopus

Search no	Query	Result
**PubMed**		
#1 Population	(newborn* OR neonat*)	1,062,112
#2 Concept	(sun OR sunlight OR heliotherapy)	755,341
#3 Context	(jaundice OR hyperbilirubin*)	60,752
#4	#1 AND #2 AND #3	235
#5	#4 AND (2000/1/1:2026/4/16[pdat])	**197**
Summary	(newborn* OR neonat*) AND (sun OR sunlight OR heliotherapy) AND (jaundice OR hyperbilirubin*) Filters: from 2000/1/1 - 2026/4/16 Sort by: Most Recent<br>(("newborn*"[All Fields] OR "neonat*"[All Fields]) AND ("sunlight"[MeSH Terms] OR "sunlight"[All Fields] OR "sun"[All Fields] OR ("sunlight"[MeSH Terms] OR "sunlight"[All Fields] OR "sunlight s"[All Fields] OR "sunlights"[All Fields]) OR ("heliotherapy"[MeSH Terms] OR "heliotherapy"[All Fields])) AND ("jaundice"[MeSH Terms] OR "jaundice"[All Fields] OR "jaundiced"[All Fields] OR "jaundices"[All Fields] OR "hyperbilirubin*"[All Fields])) AND (2000/1/1:2026/4/16[pdat])<br><br>Translations<br>sun: "sunlight"[MeSH Terms] OR "sunlight"[All Fields] OR "sun"[All Fields]<br>sunlight: "sunlight"[MeSH Terms] OR "sunlight"[All Fields] OR "sunlight's"[All Fields] OR "sunlights"[All Fields]<br>heliotherapy: "heliotherapy"[MeSH Terms] OR "heliotherapy"[All Fields]<br>jaundice: "jaundice"[MeSH Terms] OR "jaundice"[All Fields] OR "jaundiced"[All Fields] OR "jaundices"[All Fields]<br>((newborn* OR neonat*)) AND ((sun OR sunlight OR heliotherapy)) AND ((jaundice OR hyperbilirubin*))<br>Limited by: Date: After 01 January 2000<br>(TITLE-ABS-KEY (newborn* OR neonat*) AND TITLE-ABS-KEY (sun OR sunlight OR heliotherapy) AND TITLE-ABS-KEY (jaundice OR hyperbilirubin*))<br>Limited by: Date: 2000 to 2026	
**ProQuest**	((newborn* OR neonat*)) AND ((sun OR sunlight OR heliotherapy)) AND ((jaundice OR hyperbilirubin*))<br>Limited by: Date: After 01 January 2000	**642**
**Scopus**	(TITLE-ABS-KEY (newborn* OR neonat*) AND TITLE-ABS-KEY (sun OR sunlight OR heliotherapy) AND TITLE-ABS-KEY (jaundice OR hyperbilirubin*))<br>Limited by: Date: 2000 to 2026	**105**
