# Supplementary material for: Cross-cultural beliefs and practices of caregivers regarding sunlight exposure for newborn jaundice: a scoping review
Source: Front Public Health. 2026 Aug 19;14:1888701. doi: 10.3389/fpubh.2026.1888701 (PMC13533943; doi:10.3389/fpubh.2026.1888701)
Supplement: Supplementary file 2 [file Data_Sheet_2.PDF]

Appendix 2. Excluded studies and reasons for exclusion

SN	Sources	Year	Authors	Title	Reason for exclusion
1	Scopus	2002	Ahlawat D.	Neonatal hyperbilirubinemia.	Commentary. No data reported.
2	Embase®	2025	Amadi H, Muoneke LSC, Meremikwu MM, Udo JJ, Jimoh A, Ochigbo SO. et al	New solar-powered device for the treatment of neonatal hyperbilirubinaemia in hardest-to-reach places tested in Nigeria	Experimental solar-powered device. Jaundice treatment without exposure to sunlight.
3	Embase®	2020	Amouzegar A, Azizi F, Ashrafivand S, Ahi Z, Saleh M, Mohaghegh S, Gargari SS.	Prevalence of calcium and vitamin D deficiency and their association with feto-maternal outcomes in a sample of Iranian pregnant women	Sunlight exposure for jaundice not investigated. Sunlight exposure for vitamin D only.
4	Embase®	2025	Asmamaw SD, Zewde TH, Teshome A, Nigussie E.	Practice and factors associated with sunlight exposure of infants among mothers in Ethiopia: a systematic review and meta-analysis	Sunlight exposure for jaundice not investigated. Sunlight exposure for vitamin D only.
5	MEDLINE(	2026	Bhutani VK, Tiribelli C.	Intervention standards for moderate "neonatal" hyperbilirubinemia: can sunlight help?	Commentary. No data reported.
6	Embase®	2016	Bhutani VK.	Filtered sunlight noninferior to conventional phototherapy	Commentary. No data reported.
7	Embase®	2024	Bhutani VK, Wong RJ, Turkewitz D, Rauch DA, Mowitz ME, Barfield WD, et al.	Phototherapy to Prevent Severe Neonatal Hyperbilirubinemia in the Newborn Infant 35 or More Weeks of Gestation: Technical Report	Sunlight exposure for jaundice not investigated.
8	Embase®	2018	Chiemelu ON, Chiamaka A, Chinwe M, Rich U.	Hyperbilirubinaemia among neonates less than two weeks of age in rural south eastern nigeria and maternal knowledge and attitudes - A pilot study	Sunlight exposure for jaundice not investigated.
9	Embase®	2018	Colbourn Tim, Mwansambo C.	Sunlight phototherapy for neonatal jaundice—time for its day in the sun?	Commentary. No data reported.
10	PubMed	2008	Csoma Z, Kemeny L, Olah J.	Phototherapy for neonatal jaundice	Commentary. No data reported.
11	Embase®	2024	De Luca D, Bhutani VK.	Advancing neonatal phototherapy after 70 years of global impact	Commentary. No data reported.
12	Global He	2020	Dong XY, Sun X, Yu Z, Han S.	Image-based neonatal hyperbilirubinemia screening after hospital discharge.	Sunlight exposure for jaundice not investigated.
13	MEDLINE(	2021	Dong XY, Wei QF, Li ZK, Gu J, Meng DH, Guo JZ, ET AL.	Causes of severe neonatal hyperbilirubinemia: a multicenter study of three regions in China	Sunlight exposure for jaundice not investigated.
14	Scopus	2016	Dussart A, De Buyst J, Djeunang C, Janssens M, Müller M.-F, Strebelle E, et al.	Kernicterus in a homebirth baby; [Ictère nucléaire dans un contexte de naissance à domicile]	Case report. Baby exposed to natural daylight in a greenhouse.
15	MEDLINE(	2016	Emokpae AA, Mabogunje CA, Imam ZO, Olusanya BO.	Heliotherapy for Neonatal Hyperbilirubinemia in Southwest, Nigeria: A Baseline Pre-Intervention Study	Sunlight exposure for jaundice not investigated.
16	Embase®	2024	Goldenhersh MA, Eidelman AI.	Neonatal Phototherapy - The Need to Measure and Document	Opinion. Sunlight exposure not reported.
17	Embase®	2021	Gottimukkala, SB, Sethuraman G, Kitchanan S, Pathak S.	Comparison of efficacy, safety & satisfaction of intermittent versus continuous phototherapy in hyperbilirubinaemic newborns ≥35 week gestation: A randomized controlled trial	Sunlight exposure for jaundice not investigated.
18	Embase®	2016	Grzybowski A, Sak J, Pawlikowski J.	A brief report on the history of phototherapy	Commentary. Data on sunlight exposure for jaundice not reported.
19	Embase®	2013	Harrison SL, Devine SG, Saunders VL, Smith AD, Buettner PG, Nowak MJ.	Changing the risky beliefs of post-partum women about therapeutic sun-exposure	Pre and post intervention study. Prevalence data not reported.
20	MEDLINE(	2013	Harrison S, Nowak M, Devine S, Saunders V, Smith A, Buettner P.	An intervention to discourage Australian mothers from unnecessarily exposing their babies to the sun for therapeutic reasons	Pre and post intervention study. Prevalence data not reported.
21	Embase®	2021	Horn D, Ehret D, Gautham KS, Soll R.	Sunlight for the prevention and treatment of hyperbilirubinemia in term and late preterm neonates	Review of intervention studies. Prevalence data not reported.
22	Embase®	2023	Inam Y, Akbar JU, Mehdi SZ, Khan AH, Haq AU, Jalal S.	Determining the Knowledge, Attitude and Practice of Mothers Regarding Neonatal Care at Department of Pediatrics Fatima Hospital, Baqai Medical University Karachi	Sunlight exposure for jaundice not investigated.
23	Embase®	2025	Jiang L, Lee Him R, Sihota D, Muralidharan O, Dominguez G, Harrison L, et al.	Supportive Care for Common Conditions in Small Vulnerable Newborns and Term Infants: The Evidence	Review of intervention studies. Prevalence data not reported.
24	MEDLINE(	2026	John DJ, John SC, Slusher T.	A novel medical device that combines filtered sunlight phototherapy and kangaroo care to treat neonatal jaundice: bench feasibility study	Experimental study on medical device. Prevalence data not reported.
25	Embase®	2003	Johnston RV, Anderson JN, Prentice C.	Is sunlight an effective treatment for infants with jaundice?	Commentary. No data reported.
26	Embase®	2019	Kala Barathi S, Surumi M.	Assess the cultural practices on newborn care among mothers residing in Kayamkulam	Sunlight exposure for jaundice not investigated.
27	MEDLINE(	2025	Kasali BA, Udeogu J, Strauss A, Wolf JM, Brearley AM, Fajolu IB, et al.	Testing a Liquid Crystal Visual Thermometer Device in Newborns and Young Infants	Sunlight exposure for jaundice not investigated.
28	Embase®	2016	Kaur J, Kaul D	Natural sunlight as source of phototherapy in neonatal hyperbilirubinemia	Commentary. No data reported.
29	Embase®	2008	Khassawneh M, Khader Y, Amarin Z, Sa'D SA, Alkafajei A.	Traditional practices for newborns care: The north of jordan perspective	Sunlight exposure for jaundice not investigated.
30	MEDLINE(	2020	Kim MS, Chung Y, Kim H, Ko DH, Jung E, Lee BS, et al.	Neonatal exchange transfusion: Experience in Korea	Sunlight exposure for jaundice not investigated.
31	Embase®	2011	Korejo HB, Bhurgr GR, Qureshi MA, Sheikh F, Dahri GM, Chohan RK.	Etiology of neonatal jaundice an experience at Tertiary Hospital	Sunlight exposure for jaundice not investigated.
32	MEDLINE(	2022	Kuniyoshi Y, Tokutake H, Takahashi N, Kamura A, Yasuda S, Tashiro M.	Regional variation in the development of neonatal hyperbilirubinemia and relation with sunshine duration in Japan: an ecological study	Sunlight exposure for jaundice not investigated.
33	MEDLINE(	2021	Li Q, Deng X, Yan J, Sun X, Dong X, Chen X, et al.	Neonatal Severe Hyperbilirubinemia Online Registry in Jiangsu Province: protocol for a multicentre, prospective, open, observational cohort study	Sunlight exposure for jaundice not investigated.
34	Global He	2004	Luo Jz, Luo Jz.	Treatment of neonate hyperbilirubinemia by TCM combined with western medicine: a clinical observation of 25 cases.	Sunlight exposure for jaundice not investigated.
35	Embase®	2022	Mabogunje CA, Olusanya BO.	Heliotherapy for Neonatal Hyperbilirubinemia: Some Clarifications and Update	Commentary on supervised filtered sunlight exposure
36	Embase®	2015	Maisels MJ.	Sister jean ward, phototherapy, and jaundice: A unique human and photochemical interaction	Commentary. No data reported.
37	Embase®	2010	Malkin R, Anand V.	A novel phototherapy device: the design community approach for the developing world	Sunlight exposure for jaundice not investigated.
38	Scopus	2011	McCandless DW.	Phototherapy for Hyperbilirubinemia	Sunlight exposure for jaundice not investigated.
39	MEDLINE(	2012	Okwundu CI, Okoromah CAN, Shah PS.	Prophylactic phototherapy for preventing jaundice in preterm or low birth weight infants	Sunlight exposure for jaundice not investigated.

40	MEDLINE(	2026	Olusanya BO, Boo NY.	Harnessing Sunlight Safely for Newborn Care: Balancing Risks and Benefits in Resource-Limited Settings	Commentary. No data reported.
41	MEDLINE(	2024	Olusanya BO, Emokpae AA, Aina OA, Imam ZO, Olaifa SM, Owolabi OB, et al.	Heliotherapy for neonates with severe-to-hazardous hyperbilirubinemia: a randomized controlled, non-inferiority trial	Supervised filtered sunlight exposure.
42	MEDLINE(	2025	Olusanya BO, Emokpae AA, Mabogunje CA.	Filtered-sunlight phototherapy for neonatal hyperbilirubinemia: context matters	Commentary. No data reported.
43	MEDLINE(	2014	Olusanya BO, Imam ZO, Mabogunje CA, Emokpae AA, Slusher TM.	Maternal satisfaction with a novel filtered-sunlight phototherapy for newborn jaundice in Southwest Nigeria	Supervised filtered sunlight exposure.
44	MEDLINE(	2026	Olusanya BO, Omololu OM, Osamebor FB, Olufosoye A, Alo T, Olaifa SM, et al.	Filtered-sunlight phototherapy for newborns with moderate-to-severe hyperbilirubinemia: a randomized trial	Supervised filtered sunlight exposure.
45	Embase®	2026	Olusanya, Bolajoko O.; Emokpae, Abieyuwa A.; Mabogunje, Cecilia Abimbola	Filtered-sunlight phototherapy for neonatal jaundice in low-resource settings: evidence for action	Commentary on supervised filtered sunlight exposure
46	Scopus	2021	Ozsahin DU, Idoko JB, Muriritirwa NT, Moro S, Ozsahin I.	Application and impact of phototherapy on infants	Sunlight exposure for jaundice not investigated.
47	Embase®	2022	Prakash R, McGuire W.	Commentary on "sunlight for the Prevention and Treatment of Hyperbilirubinemia in Term and Late-Preterm Neonates"	Commentary on supervised filtered and unfiltered sunlight exposure
48	Embase®	2024	Reddy RVG, Parisa R.	Knowledge attitude and practices in parents regarding jaundice residing in Nalgonda District of Telangana	Sunlight exposure not investigated. Home remedies of grand parents and neighbour reported.
49	MEDLINE(	2001	Salih FM.	Can sunlight replace phototherapy units in the treatment of neonatal jaundice? An in vitro study	Experimental in vitro study.
50	MEDLINE(	2024	Saloojee H.	Innovative approaches to neonatal jaundice diagnosis and management in low-resourced settings	Review. Prevalence data not reported.
51	Embase®	2016	Santhanam I, Bhat M, Kansal B, Nadig NG, Olusanya BO, Slusher TM, Mathew, Joseph L	Training pediatric emergency medicine specialists in India	Commentary. No data reported.
52	MEDLINE(	2017	Slusher TM, Day LT, Ogundele T, Woolfield N, Owa JA.	Filtered sunlight, solar powered phototherapy and other strategies for managing neonatal jaundice in low-resource settings	Review of intervention studies. Prevalence data not reported.
53	Embase®	2018	Slusher TM, Vreman HJ, Brearley AM, Vaucher YE, Wong RJ, Stevenson DK, et. al.	Filtered sunlight versus intensive electric powered phototherapy in moderate-to-severe neonatal hyperbilirubinaemia: a randomised controlled non-inferiority trial	Supervised filtered sunlight exposure.
54	MEDLINE(	2014	Slusher TM, Vreman, HJ, Olusanya BO, Wong RJ, Brearley AM, Vaucher YE, et al.	Safety and efficacy of filtered sunlight in treatment of jaundice in African neonates	Supervised filtered sunlight exposure.
55	Embase®	2015	Slusher TM, Olusanya BO, Vreman HJ, Brearley AM, Vaucher YE, Lund TC, et al.	A randomized trial of phototherapy with filtered sunlight in african neonates	Supervised filtered sunlight exposure.
56	Embase®	2013	Slusher TM, Olusanya BO, Vreman HJ, Wong RJ, Brearley AM, Vaucher YE, et al.	Treatment of neonatal jaundice with filtered sunlight in Nigerian neonates: Study protocol of a non-inferiority, randomized controlled trial	Study protocol for supervised filtered sunlight exposure
57	Embase®	2018	Slusher TM, Vreman H, Brearley A, Adeleke O, Vaucher Yvonne, Wong R, et al.	Filtered sunlight phototherapy, a novel treatment for moderate-severe hyperbilirubinemia, is no less efficacious than conventional phototherapy in neonates in Ogbomoso, Nigeria	Supervised filtered sunlight exposure.
58	Embase®	2017	Subhashini K.	Neonatal jaundice	Review of intervention studies. Prevalence data not reported.
59	Embase®	2022	Torres M, Mallikarjuna P, Goswami S, McGrath-Blagrove T, Lew L, Pinero-Bernardo S.	Maternal knowledge and attitude on neonatal jaundice and phototherapy of term newborns in an urban multiethnic community hospital	Conference poster presentation. No full text.
60	Embase®	2013	Vreman HJ, Slusher TM, Wong RJ, Schulz S, Olusanya BO, Stevenson DK.	Evaluation of window-tinting films for sunlight phototherapy	Experimental study on medical device. Prevalence data not reported.
61	Scopus	2026	Xu JJ, Li C, Zhang S-W, Hao Y, Huang H-F, Meng X, et al.	The impact of interaction between particulate matter and sunlight duration on neonatal hyperbilirubinemia	Sunlight exposure for newborn jaundice not investigated. Maternal sunlight exposure during pregnancy only.
62	Embase®	2022	Yan Q, Li D, Yin X, Jiang N, Sun N, Luo Q, et al.	Development and validation of a maternal anxiety for neonatal jaundice scale in China	Sunlight exposure for newborn jaundice not investigated.
63	**Others**	2021	Abdul-Mumin A, Owusu EA, Wondoh PM, Tabiri S.	Maternal Knowledge and Awareness of Neonatal Jaundice in Term Neonates admitted to the Neonatal Intensive Care Unit of the Tamale Teaching Hospital. J. Med. Biomed. Sci. 8(1), 12-17.	Sunlight exposure for newborn jaundice not investigated.
64	**Others**	2013	Alparslan O, Demirel Y.	Traditional neonatal care practices in Turkey Jun 2013 JPN J NURS SCI	Sunlight exposure for newborn jaundice not investigated.
65	**Others**	2014	Aziz NA, Lee K.	Malaysian primipara's knowledge and practice on newborn care during the postnatal period. Int J Public Health Clin Sci 2014;1:132–43.	Sunlight exposure for newborn jaundice not investigated.
66	**Others**	2013	Dash MA.	Descriptive study to assess the knowledge and attitude on neonatal jaundice among the mothers in a selected village of Puducherry. Research & Reviews:J Med Health Sci 2013; 2:41-46.	Sunlight exposure for newborn jaundice not investigated.
67	**Others**	2016	Hussein H, Aziz A.	Assessment of mothers' knowledge and beliefs toward care of neonatal jaundice in pediatric teaching hospital in Holy Karbala City. Int J Sci Res 2016;6 http://www. ijsrp. org/ researchpaper-0916. php? rp= P575824	Sunlight exposure for newborn jaundice not investigated.
68	**Others**	2019	Magfouri H, Aqeel A, Maashi A, Maghfuri N, Jarad R, Kathiah AA,	Almudeer A (2019). Mothers' perception of neonatal jaundice in Jazan Region, KSA. J. Clin. Neonatol. 8(2), 116.	Sunlight exposure for newborn jaundice not investigated.
69	**Others**	2014	Nurcan Özyazıcıoğlu, Sevinç Polat.	Traditional practices frequently used for the newborn in Turkey: A literature review Jul 2014; Indian Journal of Traditional Knowledge, Vol 13(3); July 2014; 445-452 [Full text available]	Sunlight exposure for newborn jaundice not investigated.
70	**Others**	2014	Rama R, Gopalakrishnan S, Udayshankar PM.	Assessment of knowledge regarding new-born care among mothers in Kancheepuram district, Tamil Nadu. Int J Community Med Public Health 2014;1:58–63.	Sunlight exposure for newborn jaundice not investigated.
71	**Others**	2011	Rodrigo BK, Cooray G.	The knowledge, attitude & behaviour on neonatal jaundice of postnatal mothers in Provincial General Hospital, Badulla. Sri Lanka J Child Health 2011; 40:164–168. [Google Scholar]	Sunlight exposure for newborn jaundice not investigated.
