# Supplementary material for: Cross-cultural beliefs and practices of caregivers regarding sunlight exposure for newborn jaundice: a scoping review
Source: Front Public Health. 2026 Aug 19;14:1888701. doi: 10.3389/fpubh.2026.1888701 (PMC13533943; doi:10.3389/fpubh.2026.1888701)
Supplement: Supplementary file 3 [file Data_Sheet_3.PDF]

Appendix 3. Included studies and sources (n=109)

SN	Database	Country	Year	Citations in alphabetical order of first author's name
1	Others	Nigeria	2023	Adamu A, Isezuo KO. Neonatal Jaundice; Evaluating Knowledge and Practice Among Mothers Attending Immunisation Clinics of Secondary Health Care Facilities of Sokoto Metropolis. J Med Bas Sci Res. 2023;4(1-2):103-112. DOI: 10.5281/zenodo.7744233
2	Others	Nigeria	2015	Adebami OJ. Assessment of knowledge on causes and care of neonatal jaundice at the Nigerian primary and secondary health institutions. Int J Res Med Sci. 2015 Oct;3(10):2605-2612
3	Others	Malaysia	2016	Adeeb MNA, Kumarasamy K, Hamid SA, Mahat NA, Arumugam K, Shukor SHA. Knowledge and Attitude of Neonatal Jaundice – Orang Asli Perspective. J Sains Kesihatan Malaysia. 2016;14(2): 65-68. DOI : http://dx.doi.org./10.17576/JSKM-2016-1402-08
4	Others	Nigeria	2018	Adigun AS, Olabisi AP, Ogbeye GB, Kehinde A. Newborn Care Practices and Knowledge of Risk Factors Associated with Neonatal Mortality among Postnatal Mothers in Ibadan. Int J Caring Sciences. 2018;11 (2):1050-1058.
5	Others	Bangladesh	2022	Afroze S, Parvin R, Sudha RC, Abiduzzaman MF, Ehtesham Kabir ANM. Mothers' Knowledge on Neonatal Jaundice attending a Neonatal Intensive Care Unit of a Tertiary Care Hospital in Dhaka, Bangladesh. Inter J Hum Health Sci 2022;6(3):304-308. DOI: http://dx.doi.org/10.31344/ijhhs.v6i3.463
6	Others	India	2017	Aggarwal B, Agrawal A, Chaudhary P, Gupta G, Rana S, Gupta S. Neonatal Jaundice: Knowledge, attitude, beliefs, and practices of postnatal mothers in a tertiary care hospital in Uttarakhand, India. Indian J Child Health 2017;4(4):603-608.
7	Others	Nigeria	2025	Ajator CC, Okolie UR, Adenyi JM, et al. The Use of Unconventional Methods in The Treatment of Neonatal Jaundice Among Mothers Who Are Traders in Nkwo Nnewi, Anambra State, Nigeria. Global Professionals Multidisciplinary Practices Journal. 2025, 2(3):26-39
8	Others	Iraq	2022	Al-ezzi Jİ, Shareef AJ, Ali MH. Neonatal jaundice: knowledge, practice, and attitude among primigravida women. Genel Tip Derg. 2022;32(5):530-533. https://doi.org/10.54005/geneltip.1142356
9	Others	Iraq	2025	Al-Hadeethi EMT, Noaman AM, Rajab AS, Younis MA. Knowledge of mothers regarding traditional methods of neonatal jaundice treatment. Gomal Journal of Medical Sciences. 2025;23(1), 28-33.
10	Others	Jordan	2017	Al-Sagarat AY, Al-Kharabsheh A. Afr J Tradit Complement Altern Med. 2017;14 (1): 1-9. doi:10.21010/ajtcam.v14i1.1
11	Others	Iraq	2020	Al-ZAmili AH, Saadoon ZA. Knowledge, Attitude and Practice of Mothers to Neonatal Jaundice. Medico-legal Update, January-March 2020, Vol.20, No. 1. DOI:10.37506/v20/i1/2020/mlu/194442
12	PubMed	Turkey	2006	Aladag N, Filiz TM, Topsever P, Gorpelioglu S. Parents' knowledge and behaviour concerning sunning their babies; a cross-sectional, descriptive study. BMC Pediatr. 2006 Oct 31;6:27. doi: 10.1186/1471-2431-6-27. PMID: 17076884; PMCID: PMC1634747.
13	Others	Saudi Arabia	2023	Alfaifi JA, Alqahtani YA, Alqahtani MM, Alqarni A, Alshahrani A, Alshomrani RA. Knowledge and Attitudes of Parents Regarding Neonatal Jaundice in Bisha City, Saudi Arabia. Cureus. 2023; 15(9): e44916. DOI 10.7759/cureus.44916
14	Others	Saudi Arabia	2018	Alfouwais NM, Seada LS, Alahmadi RY, Alassiri AA, Alenazi AA, Aljuaeed MS. Assessment of Knowledge, Attitude and Practice of Saudi Parents towards Neonatal Jaundice (NNJ): A Cross-sectional Study. Egypt J Hosp Med. 2018;70(9):1686-1694. DOI: 10.12816/0044688
15	Others	Pakistan	2025	Altaf M, Rind KH, Noreen R, Rind NA, Shar AH, Ghulam Murtaza Khuhro GM. Bilirubin Profiles and Symptomatology in Neonates: Unraveling Gender and Location Specific Trends in Shaheed Benazirabad. J Liaquat Uni Med Health Sci 2025;1-15. doi: 10.22442/jlumhs.2025.01357
16	Others	Ghana	2019	Amegan-Aho KH, Segbefia CI, Glover NDO, Ansa GA, Afaa TJ. Neonatal Jaundice: awareness, perception and preventive practices in expectant mothers. Ghana Med J. 2019 Dec;53(4):267-272. doi: 10.4314/gmj.v53i4.3. PMID: 32116337; PMCID: PMC7036439.
17	Others	Ethiopia	2017	Anmut W, Fekecha B, Demeke T. Mother's knowledge and Practice About Neonatal Danger Signs and Associated Factors in Wolkite Town, Gurage Zone, SNNPR, Ethiopia, 2017. J Biomedical Sci. 2017, Vol. 6 No 4:33
18	PubMed	India	2023	Arumugam L, Kamala S, Ganapathy K, Srinivasan S. Traditional newborn care practices in a tribal community of Tamilnadu, South India: A mixed methods study. Indian J Community Med 2023;48:131-6. DOI:10.4103/ijcm.ijcm_498_22
19	PubMed	Ethiopia	2022	Ashebir YG, Sebsibe GT, Gela D, Kebede MA. Attitudes of mothers attending public hospitals in Addis Ababa, Ethiopia, to neonatal sunlight exposure: a cross-sectional study. BMJ Paediatr Open. 2022 Sep;6(1):e001554. doi: 10.1136/bmjpo-2022-001554. PMID: 36645760; PMCID: PMC9454020.
20	Others	Ghana	2024	Asiedu C, Edzeani EA. Assessing the Awareness and Perception of Neonatal Jaundice among Expectant Mothers in Ghana. J Pediatr Neonatal. 2024; 6(2): 1-6.
21	Others	India	2019	Barathi KS, Surumi M. Assess the Cultural Practices on Newborn Care among Mothers Residing in Kayamkulam. J. Pharm. Sci. Res. 2019;11(5):1676-1678
22	Others	Nigeria	2022	Barclay E, Ojo I, Hake A, et al. Neonatal Jaundice: Knowledge and Practices of Healthcare Providers and Trainees in Southwest Nigeria. Am J Trop Med Hyg. 2022 Jun 27;107(2):328-335. doi: 10.4269/ajtmh.21-0588. PMID: 35895425; PMCID: PMC9393447.
23	Others	Ghana	2024	Boateng K-HT, Sarah BA, Asirifi SKA. (2024). Awareness and Knowledge Level of Puerperal Mothers on Neonatal Jaundice: A Qualitative Study in Northern Ghana. Asian J Pediatr Research. 2024;14(6):45–60.
24	PubMed	Malaysia	2011	Boo NY, Gan CY, Gian YW, Lim KS, Lim MW, Krishna-Kumar H. Malaysian mothers' knowledge & practices on care of neonatal jaundice. Med J Malaysia. 2011 Aug;66(3):239-43. PMID: 22111448.
25	Others	USA	2010	Brethauer M, Carey L. Maternal experience with neonatal jaundice. MCM Am J Matern Child Nurs. 2010 Jan - Feb; 35(1): 8-14.
26	Others	China	2006	Chen WX, Wong VC, Wong KY. Neurodevelopmental outcome of severe neonatal hemolytic hyperbilirubinemia. J Child Neurol. 2006 Jun;21(6):474-9. doi: 10.1177/08830738060210061301. PMID: 16948930.
27	Others	Nigeria	2023	Chime OH, Eneh CI, Asinobi IN, et al. Caregivers perception of common neonatal illnesses and their management among rural dwellers in Enugu state, Nigeria: a qualitative study. BMC Public Health. 2023 Apr 11;23(1):665. doi: 10.1186/s12889-023-15582-2. PMID: 37041538; PMCID: PMC10088208.
28	Others	Turkey	2009	Cakmak A, Ertem M, Zeyrek D, Atas A, Karazeybek H. Maternal beliefs and attitudes concerning neonatal jaundice in southeast Turkey. Turkiye Klinikleri Journal of Medical Science 2009;29:810–815.
29	Others	Ghana	2025	Cudjoe FK, Mohammed AS, Yeboah D, Gmanyami JM, Yakong VN, Amoakoh MY. Knowledge, attitude and practice of puerperal and expectant mothers on neonatal jaundice in selected health facilities in Tamale metropolis, Ghana. medRxiv 2025.08.13.25333555; doi: https://doi.org/10.1101/2025.08.13.25333555
30	Others	UK	2026	Dann SA, Kenyon S, Adams D, et al. Understanding community detection, testing and management of neonatal jaundice in term infants in the UK: a mixed methods study. Midwifery. 2026 May;156:104738. doi: 10.1016/j.midw.2026.104738. Epub 2026 Feb 12. PMID: 41712970.
31	Embasse	Nepal	2016	Dharel D, Bhattarai A. Maternal Perception about Neonatal Jaundice in Eastern Nepal: A Qualitative Study. Health Prospect. 2016; 15(3):
32	Others	Ghana	2023	Donkor DR, Ziblim SD, Dzantor EK, Asumah MN, Abdul-Mumin A. Neonatal Jaundice Management: Knowledge, Attitude, and Practice Among Nurses and Midwives in the Northern Region, Ghana. SAGE Open Nurs. 2023 Jul 13;9:23779608231187236. doi: 10.1177/23779608231187236. PMID: 37528906; PMCID: PMC10387689.
33	Others	Israel	2025	Dotan M, Kovalski Y, Osovsky D, Levine H, Osovsky M. Pediatricians' knowledge and perception of the care of the infant with jaundice. J Pediatr Neonat Individual Med. 2025;14(1):e140111. doi:10.7363/140111.
34	Others	Vietnam	2025	Dung NT, Tam LT, Giang DTT, Tuyen DQ. Mother's Practices on Newborn Care with Jaundice at the Mountain General Hospital, Vietnam. Recent Adv Clin Trials. 2025; 5(5): 1-5.
35	Others	Nigeria	2013	Egube BA, Ofili AN, Isara AR, Onakewhor JU. Neonatal jaundice and its management: knowledge, attitude, and practice among expectant mothers attending antenatal clinic at University of Benin Teaching Hospital, Benin City, Nigeria. Niger J Clin Pract. 2013 Apr-Jun;16(2):188-94. doi: 10.4103/1119-3077.110147. PMID: 23563460.
36	Others	Nigeria	2015	Ekwochi U, Osuorah DI, Ndu IK, et al. Neonatal Jaundice: Perception and Care Seeking Behaviors among Mothers/Caregivers in a Developing Setting. Enliven: Pediatr Neonatal Biol. 2015;2(5): 009.
37	Others	Saudi Arabia	2022	Elghamrawy T, Alabdullah DW, Abukhaled JK, Aleban SA, Almalki MS, Alzahrani AA, Alkadi AN. Knowledge, attitude, and practice of neonatal jaundice: a cross-sectional study in adult population in Saudi Arabia. Int J Med Dev Ctries 2022;6(10):1269–1275. https://doi.org/10.24911/IJMDC.51-1662152312
38	Others	Egypt	2023	Elsakka EAR, Elzlbany GAM, Elsaeed BIK. Mothers' Knowledge and Caring Practices for Neonatal Physiological Jaundice: a multisite cross-sectional study in El-Beheira Governorate, Egypt. Egyptian Journal of Health Care (EJHC).2023;14(3):1212-1223
39	PubMed	Nigeria	2009	Eneh AU, Ugwu RO. Perception of neonatal jaundice among women attending children out patient and immunization clinics of the UPTH Port Harcourt. Niger J Clin Pract. 2009 Jun;12(2):187-91. PMID: 19764672.
40	PubMed	Nigeria	2022	Esan DT, Muhammad F, Ogunkorode A, Obialor B, Ramos C. Traditional beliefs in the management and prevention of neonatal jaundice in Ado-Ekiti, Nigeria. Enferm Clin (Engl Ed). 2022 Jun;32 Suppl 1:S73-S76. doi: 10.1016/j.enfcle.2021.09.006. PMID: 35688569.
41	Others	Nigeria	2014	Ezeaka CV, Ugwu RO, Mukhtar-Yola M, Ekure EN, Olusanya BO. Pattern and predictors of maternal care-seeking practices for severe neonatal jaundice in Nigeria: a multi-centre survey. BMC Health Serv Res. 2014 Apr 28;14:192. doi: 10.1186/1472-6963-14-192. PMID: 24774506; PMCID: PMC4032169.
42	Others	Nigeria	2016	Ezeaka VC, Ekure EN, Fajolu IB, Ezenwa BN, Akintan PE. Mothers' perception of neonatal jaundice in Lagos, Nigeria: An urgent need for greater awareness. S Afr J Child Health 2016;10(4):227-230. DOI:10.7196/SAJCH.2016.v10i4.1190
43	Others	Nigeria	2024	Ezeogu J, Asinobi K, Okeji CN, Chimah CT. Jaundice and Treatment Options: Knowledge, Views and Current Practices among Caregivers of Children Attending a Teaching Hospital in Owerri, Nigeria. J. Adv. Med. Med. Res. 2024;36(5):65-72.
44	Others	Brazil	2025	Fajardo AD, Pfitzer C, Rudolf E, et al. Assessment of Medical Team Knowledge on Sun Exposure and Neonatal Jaundice in a Hospital in Southern Brazil. Resid Pediatr. 2025;15(3). DOI: 10.25060/residpediatr-2025.v15n3-1350.
45	Others	Congo	2023	Fanello C, Lee SJ, Bancone G, et al. Prevalence and Risk Factors of Neonatal Hyperbilirubinemia in a Semi-Rural Area of the Democratic Republic of Congo: A Cohort Study. Am J Trop Med Hyg. 2023 Sep 5;109(4):965-974. doi: 10.4269/ajtmh.23-0293. PMID: 37669757; PMCID: PMC10551084.
46	Others	Egypt	2018	Farouk OM, Bebars GM, Ali AS, Tawfik AH. Mothers' Knowledge and Attitude Regarding Neonatal Hyperbilirubinemia. Minia Scientif Nurs J. 2018; 4(1): 22-31. doi: 10.21608/msnj.2018.187745.
47	PubMed	India	2024	Gaur R, Kumar R, Kaur N, Kumar M, Das S, Patel RB. Cultural practices and beliefs followed for new born care in Santhal pargana - A cross sectional study. J Family Med Prim Care 2024;13:3150-5.
48	PubMed	Nigeria	2015	Goodman OO, Kehinde OA, Odugbemi BA, Femi-Adebayo TT, Odusanya OO. Neonatal Jaundice: Knowledge, Attitude and practices of mothers in Mosan-Okunola community, Lagos, Nigeria. Niger Postgrad Med J 2015;22:158-63.
49	Others	Iraq	2019	Hameed NN, Abdul-Hussain NJ, Ahmed ME, Kadhim NH. Assessment of mothers' knowledge, practices, and beliefs toward home management of neonatal jaundice in two pediatric teaching hospitals. J Fac Med Baghdad 2019;61(3):119-127.DOI: https://doi.org/ 10.32007/jfacmedbagdad.613,41710
50	Others	Saudi Arabia	2022	Hanif I, Hussain S, Alanazi WKJ, Alanazi FAF, Alanazi AAQ, Alanazi ZDR. Assessment of parents' awareness level regarding neonatal jaundice in Arar, KSA. Med Sci. 2022;26(125):1-8. https://doi.org/10.54905/disssi/v26i125/ms317e2388
51	PubMed	Australia	2002	Harrison S, Hutton L, Nowak M. An investigation of professional advice advocating therapeutic sun exposure. Aust N Z J Public Health. 2002 Apr;26(2):108-15. doi: 10.1111/j.1467-842x.2002.tb00901.x. PMID: 12054327.
52	Global Health	Australia	2013	Harrison S, Nikles J, Nowak M. Don't burn the baby: Advice from Australian nurses recommending therapeutic sun exposure during infancy. Open Journal of Preventive Medicine, 2013;3:212-218. doi: 10.4236/ojpm.2013.32029.
53	Others	Pakistan	2025	Hassan M, Ali K, Mahar AH, Ahmed T, Memon S, Ali Z, Shams A. Neonatal hyperbilirubinemia: investigating the relationship between risk factors and jaundice. Frontier Med Health Res. Zenodo; 2025. DOI 10.5281/zenodo.17034015.
54	Global Health	Bangladesh	2017	Huq S, Hossain SM, Haque SMT, Tarafder MA. Knowledge Regarding Neonatal Jaundice Management among Mothers: A Descriptive Study Done In a Tertiary Level Hospital of Dhaka City. AKMMC J 2017; 8(2) : 121-127.

55	Others	Nigeria	2020	Iliyasu Z, Farouk Z, Lawal A, Bello MM, Nass NS, Aliyu MH. Care-seeking behavior for neonatal jaundice in rural northern Nigeria. Public Health Pract (Oxf). 2020 Apr 11;1:100006. doi: 10.1016/j.puhip.2020.100006. PMID: 36101691; PMCID: PMC9461625.
56	Others	Mexico	2024	Jiménez-Díaz G, Aune A, Elizarrarás-Rivas J, Gierman LM, Keitsch M, Marcuzzi A, Infanti JJ. Neonatal jaundice detection in low-resource Mexican settings: possibilities and barriers for innovation with mobile health. BMC Health Serv Res. 2024 May 28;24(1):671. doi: 10.1186/s12913-024-11141-6. PMID: 38807158; PMCID: PMC11134921.
57	Others	Iraq	2016	Karim KH. Home Practices of Kurdish Mothers Regarding Management of Jaundice among Newborns in Erbil City. Int J Sci Res Pub. 2016;6(9): 383-388. www.ijsrp.org
58	Others	India	2021	Khound M. Knowledge, Attitude and Practice of mothers regarding Neonatal Jaundice: A Hospital-based Observational Study. Int J Innovative Sci Res Tech. 2021;6(10:458-460
59	Others	USA	2008	Kuzniewicz MW, Escobar GJ, Wi S, Liljestrand P, McCulloch C, Newman TB. Risk factors for severe hyperbilirubinemia among infants with borderline bilirubin levels: a nested case-control study. J Pediatr. 2008 Aug;153(2):234-40. doi: 10.1016/j.jpeds.2008.01.028. Epub 2008 Mar 21. PMID: 18534217; PMCID: PMC3142930.
60	Others	Vietnam	2014	Le LT, Partridge JC, Tran BH, Le VT, Duong TK, Nguyen HT, Newman TB. Care practices and traditional beliefs related to neonatal jaundice in northern Vietnam: a population-based, cross-sectional descriptive study. BMC Pediatr. 2014 Oct 14;14:264. doi: 10.1186/1471-2431-14-264. PMID: 25316215; PMCID: PMC4287314.
61	Others	China	2008	Liu Q. Preventive effect of Chinese herbal medicine soaking bath plus sunbathing on neonatal jaundice [in Chinese]. Chin J Misdiagnostics 2008; 8: 7137–38.
62	Others	Uruguay	2025	Lores P, Beñarán V, Dodino A, Gonçalves G, Pigazzini A, Freire T. Understanding the maternal perception of neonatal jaundice: Insights from a hospital in Uruguay. Midwifery. 2025 Nov;150:104607. doi: 10.1016/j.midw.2025.104607. Epub 2025 Sep 12. PMID: 40966885.
63	Others	Saudi Arabia	2024	Mahmoud KH, Alotaibi FA, Alsahli AM, et al. The Prevalence, Practice, and Attitude Toward Neonatal Jaundice Among Saudi Mothers. Cahiers Magellanes-NS. 2024;6(2):8192-8214. http://magellanes.com/
64	Others	Ethiopia	2023	Mesele M, Abebe K, Dessu S, et al. Mothers' health care seeking behavior for neonatal danger sign in southern Ethiopia: Community based cross–sectional study. PLoS ONE 2023;18(7): e0280993. https://doi.org/10.1371/journal.pone.0280993
65	Others	India	2024	Metta S, AASALA ST, SINGH AK, BHAVISHA B, KAPPALA A, KHOKLE S. A prospective observational exploration of maternal awareness of neonatal jaundice and risk assessment. QUANTUM JOURNAL OF MEDICAL AND HEALTH SCIENCES 3(3): 37-55.
66	Embasse	Egypt	2016	Moawad EMI, Abdallah EAA, Ali YZA. Perceptions, practices, and traditional beliefs related to neonatal jaundice among Egyptian mothers: A cross-sectional descriptive study. Medicine. 2016;95:36. http://dx.doi.org/10.1097/MD.0000000000004804
67	Others	Sudan	2022	Mohammed Ahmed NSO, Al- Gamar EA. Mother's knowledge and attitude regarding recognition of neonatal signs of danger. Sudan J Paediatr. 2022;22(1):27–35. https://doi.org/10.24911/SJP.106-1524987299.
68	Others	Iraq	2021	Mohammed Kassim N, Fadhil Obaid A, Abdulameer Abdulrasol Z. The Traditional Practices of Mothers in Caring of Neonates Affected by Hyperbilirubinemia. Iranian Rehabilitation Journal. 2021; 19(4):433-440. http://dx.doi.org/10.32598/irj.19.4.1621.1
69	Others	Iraq	2021	Mohsin MF, Obaid SS, Almhanna MGH. Knowledge and attitude of mothers towards neonatal jaundice in Karbalaa Teaching Hospital for Children. Karbala J Med. 2021;14(2):2534-2541. https://doi.org/10.70863/karbalajm.v14i2.864
70	Others	Egypt	2019	Mostafa MA, Kamal NM , Eltahe S, et al. Knowledge of Neonatal Hyperbilirubinemia Among Primary Health Care Physicians: A Single-Centre Experience. Clinical Medicine Insights: Pediatrics. 2019;13:1-5. DOI: 10.1177/1179556518824375
71	Others	Saudi Arabia	2024	Mudhol B, Jha P, Reddy NV, et al. Attitude and Practice Regarding Sunning of Babies among Mothers of Selected Communities of Selected Area. Nanotechnology Perceptions. 2024;20(1):167–175. www.nano-ntp.com
72	Others	Uganda	2024	Nalubega P, Ssali A, Namugumya R, et al. Community Perceptions of Neonatal Infection in Uganda. Open Forum Infect Dis. 2025 Mar 10;11(Suppl 3):S206-S211. doi: 10.1093/ofid/ofae607. PMID: 40070701; PMCID: PMC11891128.
73	Others	Malaysia	2014	Ng SY, Chong SY. What Do Mothers know about Neonatal Jaundice? Knowledge, Attitude and Practice of Mothers in Malaysia. Med J Malaysia 2014;69:252-256
74	Others	Nigeria	2015	Ogunlesi TA, Abdul AR. Maternal knowledge and care-seeking behaviors for newborn jaundice in Sagamu, Southwest Nigeria. Niger J Clin Pract. 2015 Jan-Feb;18(1):33-40. doi: 10.4103/1119-3077.146976. PMID: 25511341.
75	Others	Nigeria	2013	Okperi BO. Neonatal jaundice and birth asphyxia as major causes of cerebral palsy in Nigeria: are doctors' wrong beliefs and practices part of the problem? Int J Med Biomed Res. 2013;2(3):226 -230.
76	Others	Nigeria	2014	Okposio MM, Adebanjo EO. Evaluation of knowledge and perception of newborn jaundice among parturient mothers in a secondary health care centre in the Niger Delta region of Nigeria. Bangladesh J Med Science. 2014;13(3):292-297. DOI: http://dx.doi.orghttp://dx.doi.org/10.3329/bjms.v13i3.16521
77	Others	Nigeria	2020	Olatunde OE, Christianah OA, Olarinre BA, et al. Neonatal Jaundice: Perception of Pregnant Women Attending Antenatal Clinic at a Tertiary Hospital in Southwest, Nigeria. Glob Pediatr Health. 2020 Dec 20;7:2333794X20982434. doi: 10.1177/2333794X20982434. PMID: 33426182; PMCID: PMC7756043.
78	Others	Nigeria	2016	Onyearugha CN, Chapp-Jumbo A, George IO. Neonatal jaundice: evaluating the knowledge and practice of expectant mothers in Aba, Nigeria. J Health Sci Res. 2016;1:42-47.
79	PubMed	Nigeria	2017	Orimadegun AE, Ojebiyi AO. Primary health workers' knowledge and practices relating to neonatal jaundice in Ibadan, Nigeria. Afr J Prm Health Care Fam Med. 2017;9(1), a1081. https://doi.org/10.4102/phcfm.v9i1.1081
80	Others	Nigeria	2022	Owolabi WF, Akinyemi OO, Owolabi BA. Perception of Mothers Towards Neonatal Jaundice in Ekiti State University Teaching Hospital, Ado-Ekiti, Ekiti State. 2022;4(7):1-13
81	Others	USA	2006	Petrova A, Mehta R, Birchwood G, Ostfeld B, Hegyi T. Management of neonatal hyperbilirubinemia: pediatricians' practices and educational needs. BMC Pediatr. 2006 Mar 6;6:6. doi: 10.1186/1471-2431-6-6. PMID: 16519797; PMCID: PMC1450287.
82	Others	Singapore	2007	Poon WB, Ho WL, Yeo CL. Survey on parenting practices among Chinese in Singapore. Singapore Med J. 2007 Nov;48(11):1006-11. PMID: 17975690.
83	Others	Thailand/Myanmar	2017	Prins TJ, Trip-Hoving M, Paw MK, et al. A Survey of Practice and Knowledge of Refugee and Migrant Pregnant Mothers Surrounding Neonatal Jaundice on the Thailand-Myanmar Border. J Trop Pediatr. 2017 Feb;63(1):50-56. doi: 10.1093/tropej/fmw055. Epub 2016 Aug 29. PMID: 27576869; PMCID: PMC5301969.
84	Others	India	2015	Purani C, Patel P, Gupta K, Mehariya KM, Holda A. Knowledge, awareness and practice of postnatal care among mothers. Indian J Child Health. 2015;2(2):83-85.
85	Others	Pakistan	2022	Qumer M, Fatima T, Albert A, Riaz S. Knowledge, Attitude and Practices of Women Towards the Neonatal Jaundice in Pakistan. Merit Res. J. Med. Med. Sci. 2022; 10(4):125-134. DOI: 10.5281/zenodo.6478595
86	Others	Iran	2014	Rabiyeepoor S, Gheibi S, Jafari S. To Study the Knowledge and Attitude of Postnatal Mothers on Neonatal Jaundice in Motahari Hospital, Iran. Clinical Medicine Research. 2014;3(1):1-5. doi: 10.11648/j.cmr.20140301.11
87	Others	Bangladesh	2023	Rahman M, Jahan F, Billah SM, et al. Feasibility and acceptability of home-based neonatal hyperbilirubinemia screening by community health workers using transcutaneous bilimeters in Bangladesh. BMC Pediatr. 2023 Apr 3;23(1):155. doi: 10.1186/s12887-023-03969-4. PMID: 37009866; PMCID: PMC10068237.
88	Others	Bangladesh	2010	Rasul CH, Hasan MA, Yasmin F. Outcome of neonatal hyperbilirubinemia in a tertiary care hospital in bangladesh. Malays J Med Sci. 2010 Apr;17(2):40-4. PMID: 22135536; PMCID: PMC3216155.
89	Others	UK	2019	Rennie JM, Beer J, Upton M. Learning from claims: hyperbilirubinaemia and kernicterus. Arch. Dis. Child.: Fetal Neonatal Ed. 2019;104 (2):F202–F204.
90	Embasse	India	2014	Reshma R, Sujatha R. Cultural Practices and Beliefs on Newborn Care Among Mothers in a Selected Hospital of Mangalore. Taluk.Nitte University Journal of Health Science.2015;4:21-26.
91	Others	Egypt	2026	Sabra HEA, Ahmed SM, Elsayed FEK. Assessment of Mothers' Knowledge and Practices Regarding Neonatal Jaundice. Sohag Journal of Nursing Sciences 2026;5(8):178-191.
92	Others	Pakistan	2022	Saddozai SU, Gul J, Shukat R, et al. Knowledge, attitudes, and practices (KAP) of mothers regarding neonatal jaundice at KPK Hospital, Pakistan. Pak J Med Health Sci. 2022;21(25):807-810.
93	Others	Ghana	2021	Salia SM, Afaya A, Wuni A, et al. Knowledge, attitudes and practices regarding neonatal jaundice among caregivers in a tertiary health facility in Ghana. PLoS ONE 2021;16(6): e0251846.
94	PubMed	Indonesia	2021	Sampurna MTA, Rohsiswatmo R, Primadi A, et al. The knowledge of Indonesian pediatric residents on hyperbilirubinemia management. Heliyon. 2021 Apr 6;7(4):e06661. doi: 10.1016/j.heliyon.2021.e06661. Erratum in: Heliyon. 2021 May 11;7(5):e07007. doi: 10.1016/j.heliyon.2021.e07007. PMID: 33898814; PMCID: PMC8056408.
95	Others	India	2017	Sasikala P, Jyothi C, Chandrasekhar V, Kumar C, Bhaskar S. A study on Traditional Beliefs and Practices in Newborn Care among Women Attending UHTC, NMC, Nellore A.P. Nation J Res Comm Med. 2017;6(2):120-124.
96	Others	Ghana	2022	Seneadza NAH, Insaidoo G, Boye H, et al. Neonatal jaundice in Ghanaian children: Assessing maternal knowledge, attitude, and perceptions. PLoS ONE 2022;17(3): e0264694. https://doi.org/10.1371/journal.pone.0264694
97	Embasse	Spain	2026	Sequí-Canet JM, Alba-Fernández J, del Rey-Tormos R, Angelats-Romero CM, Sequí-Sabater JM. Sunlight through residential glazing and prevention of neonatal jaundice. Annals Pediatr, March 25, 2026. DOI: 10.1016/j.anpedi.2026.504206.
98	Others	Nigeria	2020	Shehu M, Shehu H, Ubanyi TO. Analysis of Mothers' Knowledge, Beliefs and Practice towards Neonatal Jaundice in Bingham University Teaching Hospital Jos, Plateau State, Nigeria. Asian J Pediatr Res. 2020;4(2): 9-19.. DOI: 10.9734/AJPR/2020/v4i230144
99	Embasse	Rwanda	2021	Sheth K, Tuyisenge L, Bhutani VK. Does provider access to technology improve health care? Evidence from a national distribution of phototherapy in Rwanda. Semin Perinatol. 2021 Feb;45(1):151359. doi: 10.1016/j.semperi.2020.151359. Epub 2020 Dec 2. PMID: 33317824.
100	Scopus	Nepal	2019	Shrestha S, Maharjan S, Shrestha S, Petrini M. Knowledge about neonatal jaundice among Nepalese mothers. J BP Koirala Inst Health Sci. 2019;2(1):34-42. https://doi.org/10.3126/jbpkihs.v2i1.24965
101	Embasse	India	2025	Singh A, Kaushik SL, Gupta H. Knowledge and Awareness of Mothers Regarding Neonatal Jaundice. NeuroQuantology2025;23(6):309-317
102	Others	Jordan	2026	Tailakh SN, Basha ASH, Badran EF. Knowledge, Practices, and Attitudes of Jordanian Parents Regarding Neonatal Jaundice Associated with ABO Incompatibility: A Cross-sectional Study. Jordan J Nurs Res. 2026;5(1):74-83. DOI: https://doi.org/10.14525/JJNR.v5i1.06
103	Others	Iraq	2019	Thabit MF. Knowledge regarding neonatal jaundice among a sample of mothers attending some Primary Health Care centers /Baghdad. Diyala J Med 2019;17(1):77-83.
104	Others	India	2021	Vernekar PP, Cacodcar JA, Panandikar M, Almeida I. A Study on Traditional Beliefs and Practices during the Postpartum Period among Mothers at a District Hospital in Goa. Int J Preven Curat Comm Med. 2021;7(3):1-11.
105	Others	Nigeria	2021	West BA, Aitafo JE, Altraide BO. Neonatal Jaundice: Knowledge, Attitude and Practice among Pregnant Women Attending the Antenatal Clinic of Rivers State University Teaching Hospital, Nigeria. Inter J Trop Dis Health. 2021;42(23): 1-11. DOI: 10.9734/IJTDH/2021/v42i2330559
106	PubMed	Ghana	2024	Wolski A, Moyer CA, Amoah R, Otoo B, Kaselitz E, Bakari A. Community health worker knowledge and perceptions of neonatal jaundice in Kumasi, Ghana. Res Sq [Preprint]. 2024 Jul 29:rs.3.rs-4662211. doi: 10.21203/rs.3.rs-4662211/v1. PMID: 39149478; PMCID: PMC11326413.
107	Others	Pakistan	2016	Yaqub A, Safdar F, Ghani Z. To Assess the Knowledge of Mothers Regarding Neonatal Jaundice Presenting to Rawal Institute of Health Sciences, Islamabad. Isra Med J. 2016; l8(1).
108	Others	Ghana	2025	Zakaria A-FS, Boampong OL, Kyiu C, et al. Maternal knowledge, attitudes, and determinants of practices in neonatal jaundice care at Tamale Teaching Hospital. J Neonatal Nurs 2025; 31(6): 101729
109	Embasse	China	2015	Zhang L, Hu P, Wang J, Zhang M, Zhang QL, Hu B. Prenatal Training Improves New Mothers' Understanding of Jaundice. Med Sci Monit. 2015 Jun 8;21:1668-73. doi: 10.12659/msm.893520.
